# TD-60 links RalA GTPase function to the CPC in mitosis

**DOI:** 10.1038/ncomms8678

**Published:** 2015-07-09

**Authors:** Diana Papini, Lars Langemeyer, Maria A. Abad, Alastair Kerr, Itaru Samejima, Patrick A. Eyers, A. Arockia Jeyaprakash, Jonathan M. G. Higgins, Francis A. Barr, William C. Earnshaw

**Affiliations:** 1 Wellcome Trust Centre for Cell Biology, Institute of Cell Biology, University of Edinburgh, Michael Swann Building, Kings Buildings, Max Born Crescent, Edinburgh EH9 3BF, UK; 2 Institute for Cell and Molecular Biosciences (ICaMB), Newcastle University, Medical School, Framlington Place, Newcastle upon Tyne NE2 4HH, UK; 3 Department of Biochemistry, University of Oxford, South Parks Road, Oxford OX1 3QU, UK; 4 Department of Biochemistry, Institute of Integrative Biology, University of Liverpool, Crown St, Liverpool L69 7ZB, UK

## Abstract

TD-60 (also known as RCC2) is a highly conserved protein that structurally resembles the Ran guanine exchange factor (GEF) RCC1, but has not previously been shown to have GEF activity. TD-60 has a typical chromosomal passenger complex (CPC) distribution in mitotic cells, but associates with integrin complexes and is involved in cell motility during interphase. Here we show that TD-60 exhibits GEF activity, *in vitro* and in cells, for the small GTPase RalA. TD-60 or RalA depletion causes spindle abnormalities in prometaphase associated with abnormal centromeric accumulation of CPC components. TD-60 and RalA apparently work together to contribute to the regulation of kinetochore–microtubule interactions in early mitosis. Importantly, several mitotic phenotypes caused by TD-60 depletion are reverted by the expression of a GTP-locked mutant, RalA (Q72L). The demonstration that a small GTPase participates in the regulation of the CPC reveals a level of mitotic regulation not suspected in previous studies.

## Abstract (web summary)

TD-60 (RCC2) structurally resembles a guanine nucleotide exchange factor (GEF), but its target GTPase was unknown. Here Papini *et al.* show that TD-60 is a GEF for RalA, and that RalA helps to regulate the chromosomal passenger complex and kinetochore–microtubule interactions in mitosis.

## Graphical abstract

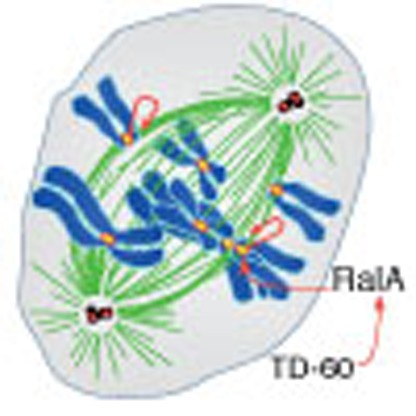

TD-60 (Telophase Disk-60), also known as RCC2, was originally identified using a human autoimmune serum that stained the anaphase spindle midzone^1^. This staining resembled that seen for the chromosomal passenger complex (CPC), a major regulator of mitosis^2^, which was originally defined based on its movement from inner centromeres in early mitosis to the spindle midzone and midbody during mitotic exit^3^.

The CPC is composed of Aurora B kinase^4^ plus an activation/targeting module consisting of inner centromere protein INCENP^3^, Survivin and Borealin/Dasra B^5^,^6^. The complex regulates key aspects of mitosis, including chromosome and spindle structure, the correction of kinetochore–microtubule attachment errors, the spindle assembly checkpoint and cytokinesis^2^. Depletion of any CPC component induces delocalization of the others and disrupts mitotic progression^5^,^7^,^8^,^9^.

Although TD-60 is not a member of the core CPC, it becomes mislocalized if CPC components are suppressed in mammalian cells^5^. Similarly, TD-60 knockdown perturbs the localization of other CPC members^10^,^11^. Furthermore, it shares a characteristic phospho-epitope with INCENP and Aurora B^12^. In *Xenopus Laevis* extracts, TD-60 depletion does not affect CPC centromeric localization, but Aurora B kinase activity is compromised^11^. TD-60 binds microtubules^10^, and can stimulate Aurora B kinase activity in the presence of microtubules *in vitro*^11^.

Cloning revealed TD-60 to be related to RCC1 (regulator of chromatin condensation 1)^10^, a guanine exchange factor (GEF) for the small GTPase Ran^13^. This led to the protein being annotated as RCC2 in databases. TD-60 binds the nucleotide-free form of Rac1 *in vitro*, and was suggested to be a Rac1 GEF^10^.

TD-60/RCC2 has also been identified in studies looking at cellular migration. It is found in pull downs of α5β1-fibronectin complexes at nodes of the interactome near Rac1, which controls cell protrusions, and Arf6, a regulator of membrane trafficking^14^. This confirmed a previous proteomic study that also found an interaction between TD-60 and Arf6 (ref. 15). The later study concluded that TD-60 is required for certain types of cellular motility, but that it does so by limiting the activity of Rac1 and Arf6, rather than acting as a GEF^14^. TD-60/RCC2 also co-immunoprecipitates with the regulator of actin filament assembly cortactin^16^. The functional significance of these interactions is not known but TD-60/RCC2, Arf6 and cortactin have all been associated with tumour invasiveness and metastasis in melanoma^17^,^18^ and other cancers^19^,^20^.

Here, we show that TD-60 is a GEF, with an unexpected substrate—the small Ras-like GTPase RalA. RalA is thought to cooperate with Arf6 in the activation of phospholipase D^21^, and is also thought to have a role in oncogenesis^17^,^22^,^23^,^24^. We report that TD-60 has GEF activity towards RalA *in vitro* and in cells; that cells depleted of TD-60 or RalA show similar mitotic phenotypes including perturbed spindles, higher microtubule density at kinetochores and increased inter-kinetochore stretch; and that this corresponds with decreased Aurora B activity at prometaphase centromeres. Importantly, wild-type (WT) Aurora B activity could be restored in cells lacking TD-60 by expressing the constitutively active GTP-locked RalA mutant Q72L. Our study links TD-60 activation of RalA with the CPC in regulating kinetochore–microtubule interactions in early mitosis.

## Results

### TD-60 is a RalA GEF

**Figure 1:**
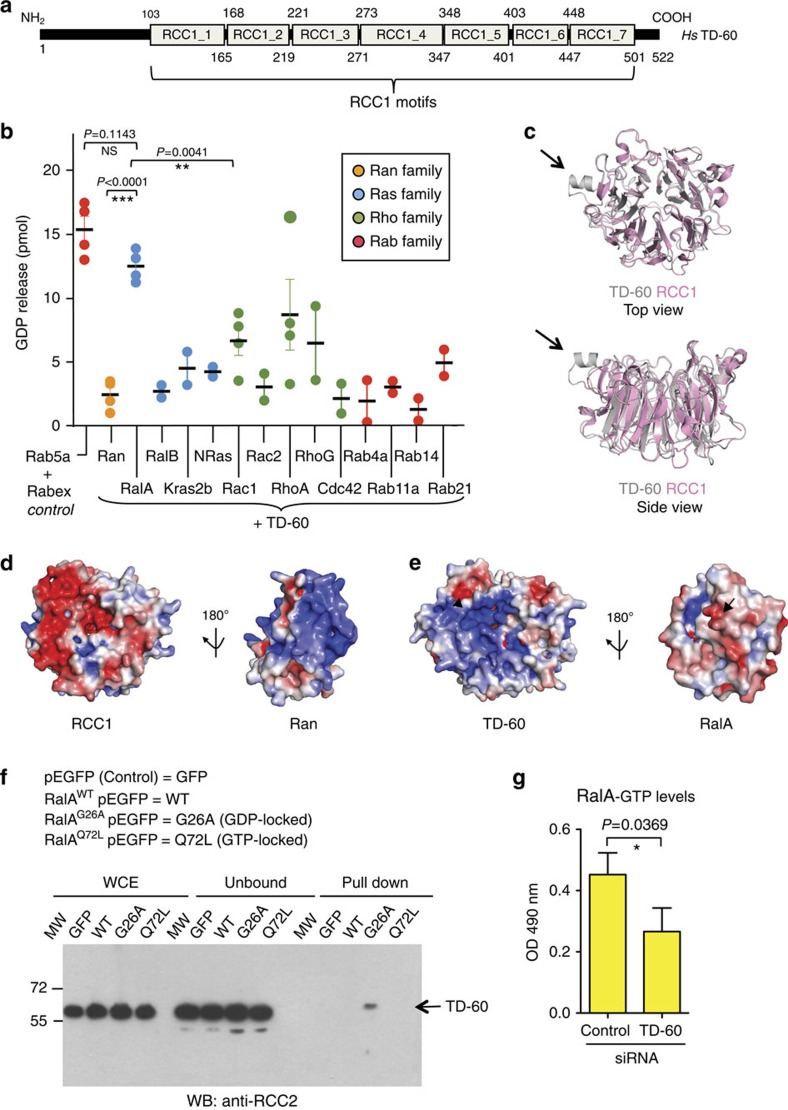
TD-60 has GEF activity towards RalA. (**a**) Schematic diagram of the human TD-60 protein showing RCC1 motif distribution. (**b**) Human TD-60 was tested against a representative panel of human small GTPase proteins using a published GDP-release assay^30^,^70^. Nucleotide exchange was calculated as pmoles of GDP released (*n*=2,4). Graph reports mean±s.e.m. Statistical significance was determined using unpaired Student's *t*-test (***P<0.0001). Additional statistical values can be found in Supplementary Table 1 (**c**) Superposition of a TD-60 structure (grey) predicted using Phyre2 ( http://www.sbg.bio.ic.ac.uk/phyre2)^34^, onto the crystal structure of RCC1 (pink—PDB id: 1I2M) suggests that the overall structure of TD-60 is similar to RCC1 with an insertion of an α-helical segment (highlighted by arrows) which is unique to higher vertebrate TD-60. (**d**) Surface representation of RCC1 and Ran highlighting the charge complementarity of their binding interfaces (both shown facing the viewer, such that a 180° flip of one of the binding partners brings the two interacting surfaces together). Blue and red represent positive and negatively charged regions, respectively. Electrostatic surface potential of RCC1 and Ran (PDB id: 1I2M) was calculated using the APBS plug-in in PyMOL (The PyMOL Molecular Graphics System, Version 1.5.0.4 Schrödinger, LLC). (**e**) Similar analysis of surface charge for TD-60 and RalA (PDB id: 2A78). (**f**) Pull-down assay reveals that TD-60 interacts specifically with RalA when bound to GDP in cells. HeLa Kyoto cells were transfected to express GFP-tagged RalA WT, G26A (GDP) and Q72L (GTP), and endogenous TD-60 was pulled down using GFP-Trap-M beads. (**g**) TD-60 depletion reduces RalA activation in cells. HeLa cells were transfected with TD-60 siRNA and RalA-GTP levels in lysates were measured by G-LISA assay. Graph reports the mean absorbance at 490 nm±s.e.m. (*n*=3), with statistical significance assessed using unpaired Student's *t*-test (*P<0.05).

TD-60 is a highly conserved protein that shares sequence similarity with RCC1 (ref. 10), particularly between amino-acid residues 100 and 500, which consist almost entirely of seven RCC1 motifs (Fig. 1a and Supplementary Fig. 1a). Because RCC1 is a GEF for the small GTPase Ran^25^,^26^, it was initially assumed that TD-60 would exhibit GEF activity, possibly for Rac1 (ref. 10). However, previous studies failed to demonstrate this GEF activity.

To characterize TD-60-associated GEF activity *in vitro*, we expressed human TD-60 containing an amino-terminal streptavidin-binding protein (SBP) tag in Sf9 cells. Cloning and expression of the human TD-60 cDNA was only possible following the replacement of a 5′ low complexity region of ∼250 bp that is unstable in *E. coli* (Supplementary Fig. 1b–d) with a sequence that was codon optimized for baculovirus expression (Supplementary Fig. 1e).

Purified SBP-TD-60 was tested for GEF activity using a GDP-release assay against a broad selection of highly purified human GTPase targets representing all GTPase superfamilies (Supplementary Fig. 2a)^27^,^28^. We have used this end-point assay in a number of previous studies to directly compare the GEF activity of a candidate regulatory factor against a panel of different GTPases under the same experimental conditions^27^,^29^. The purification of the GTPases and the assay itself have been described in detail^30^. The well-characterized GEF Rabex5, and its substrate, the GTPase Rab5a^31^, provided a positive control for GEF activity. This GEF/substrate pair catalysed the release of 15 pmol GDP under the standardized conditions used (Fig. 1b).

Unexpectedly, TD-60 displayed consistent high GEF activity (12.5 pmol GDP released) for the Ras-related protein RalA^32^,^33^ (Fig. 1b). This was not statistically different from the activity of Rabex5 towards Rab5A (*P*=0.0578; Student's *t*-test). In contrast, TD-60 exhibited little activity for expected targets from the Rho GTPase superfamily^10^, including Rac1 (for example, comparison of the Rac1 measurement versus that for RalA yielded a *P*=0.0041; Student's *t*-test; Fig. 1b and Supplementary Table 1). Despite its similarity to RCC1, TD-60 showed no activity towards Ran (comparison against the value for RalA yielded a *P*<0.0001).

An explanation for the latter result is readily apparent when one examines the electrostatic surface potential for the interface between RCC1 and Ran. We calculated a predicted TD-60 structure using the Phyre2 Server^34^, and superimposed this onto the RCC1 crystal structure (PDB id: 1I2M—Fig. 1c). TD-60 is predicted with a high level of confidence to be structurally extremely similar to RCC1, with the exception of an alpha-helical segment that is exclusive to vertebrate TD-60 (arrows, Fig. 1c)^10^. The Ran-binding region of RCC1 is predominantly negatively charged and this binds to a highly positively charged surface of Ran (Fig. 1d). In marked contrast, the corresponding surface of the TD-60 model is positively charged and could potentially dock with a negatively charged ridge on RalA (arrow, Fig. 1e).

Although GEFs, as enzymes, typically exhibit transient low-affinity interactions with their substrates, we were able to confirm the physical interaction between TD-60 and RalA *in vitro*. We did this by far-western analysis, in which purified RalA bound to purified TD-60 (Supplementary Fig. 2b). In a parallel control experiment, purified Ran did not bind to TD-60 (Supplementary Fig. 2b). To determine if TD-60 could interact with RalA also in cells, we immunoprecipitated endogenous TD-60 from HeLa cells transfected with green fluorescent protein (GFP)-tagged RalA WT, or the two mutants, RalA G26A (GDP-locked) or Q72L (GTP-locked). As detected by immunoblotting, TD-60 co-immunoprecipitated with the RalA G26A mutant (GDP-locked; Fig. 1f). A similar result was obtained in an independent repeat of this experiment (Supplementary Fig. 2d).

In addition, when we measured RalA-GTP levels in cells using a specific RalA effector protein-binding assay, cells lacking TD-60 showed a statistically significant decrease in RalA-GTP (*P*=0.0369, Student's *t*-test) versus control short interfering RNA (siRNA) cells (Fig. 1g). We conclude that, like RCC1, with which it shares sequence and likely structural similarity, TD-60 is indeed a GEF, but for RalA and not for Ran or Rho family members.

### TD-60 depletion perturbs spindles and centromeric Aurora B

TD-60 got its name from the characteristic staining of the anaphase spindle midzone observed using a human autoantibody^1^. That staining resembled the characteristic CPC localization^3^, however, TD-60 does not co-immunoprecipitate with the CPC, even though immunostaining experiments suggest some functional link with the complex^5^,^10^. In support of the conclusion that TD-60 is not a component of the CPC *in vivo*, perturbation of mitotic chromosome structure often produces very different effects on the CPC and TD-60. For example, depletion of the chromokinesin KIF4A causes a modest, but reproducible, decrease in chromosomal levels of the four core CPC subunits, whereas the levels of TD-60 show a significant increase (Supplementary Fig. 2e). We have shown previously that the individual subunits of protein complexes typically co-vary in this analysis^35^.

**Figure 2:**
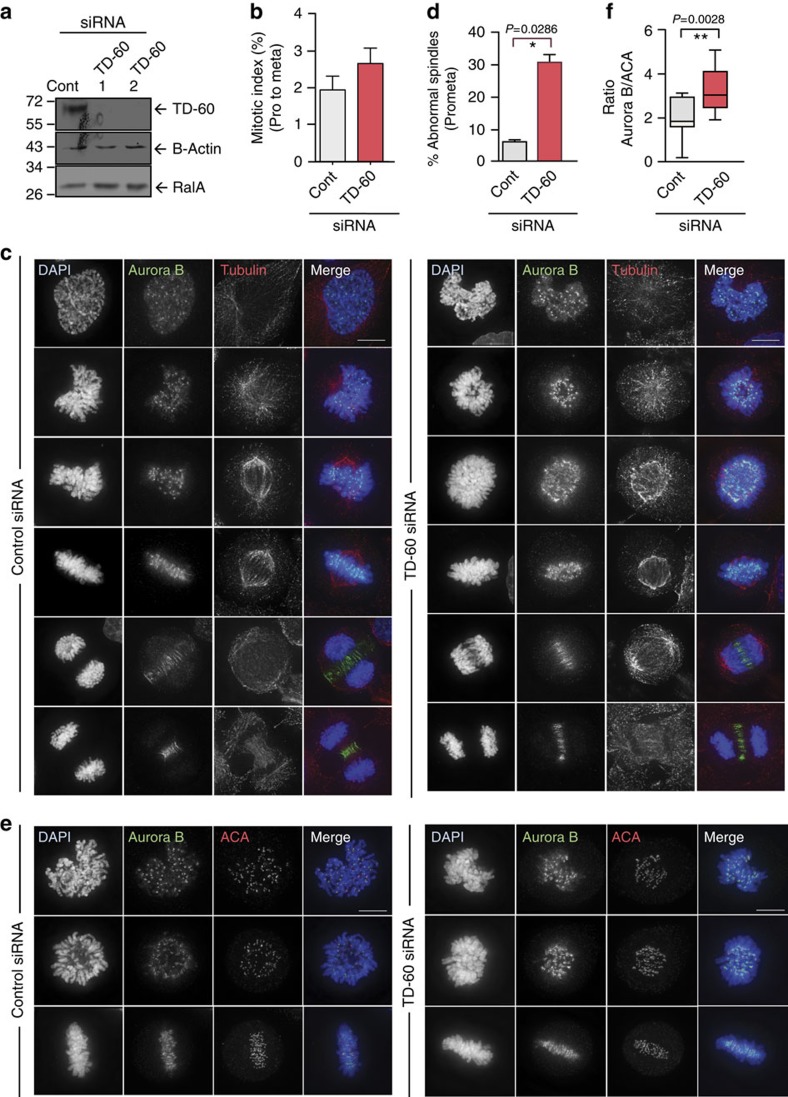
TD-60 siRNA perturbs prometaphase spindles and centromeric Aurora B. (**a**) Immunoblots of whole HeLa cell extract (WCE) transfected with siRNAs targeting two different regions of human TD-60. Immunoblot for RCC2 (TD-60) and RalA, with β-actin as a loading control. (**b**) Mitotic index of TD-60-depleted cells 48 h after transfection with siRNA is increased from prophase to metaphase. Graphs report the mean±s.e.m. (*n*=4). (**c**) Control and TD-60-depleted cells fixed with 4% PFA were stained with DAPI (blue), α-tubulin (red) and aurora B (green). Scale bar, 10 μm. (**d**) The proportion of prometaphase cells with abnormal spindle shapes (in which the poles were partly separated but nascent spindles were confined within the regions containing chromosomes) in TD-60-depleted and control cultures was determined by a blinded observer (*n*=4). In this and all subsequent figures, graphs report the mean±s.e.m., with statistical significance assessed using the Mann–Whitney *U*-test (*P<0.05). (**e**) Both prometaphase and metaphase cells show an increased level of centromeric Aurora B after 48 h TD-60 depletion. TD-60-depleted cells were fixed with 4% PFA and stained with DAPI (blue), ACA (red) and Aurora B (green). Scale bar, 10 μm. (**f**) Quantification of Aurora B intensity from the experiment illustrated in **e** using ImageJ and normalized to the ACA control (*n*=15, **P<0.005 by Mann–Whitney *U*-test). More information regarding quantification can be found in Supplementary Fig. 4b. DAPI, 4′,6-diamidino-2-phenylindole.

We used RNA interference (RNAi) to examine the role of TD-60 in mitosis in HeLa cells. TD-60 protein was significantly depleted in HeLa cells 48 h after transfection with two published TD-60-specific siRNAs (Fig. 2a)^10^. We observed a higher frequency of prophase and prometaphase cells in TD-60-depleted samples (Fig. 2b, Supplementary Fig. 3a). These results contrast with a published report in which TD-60 depletion was reported to cause a strong arrest of cells in prometaphase^10^. Importantly, in that study, cells were examined mainly during recovery from 16 h of nocodazole treatment. A more recent study from that group has reported that the depletion of TD-60 causes a decrease in mitotic entry^36^. In our study, the siRNAs were added to asynchronous cultures allowing us to follow unperturbed entry into and passage through mitosis.

The increase in cells in early mitosis following TD-60 depletion was counterbalanced by a decrease in cells in telophase and cytokinesis (Supplementary Fig. 3a). Thus, after an initial delay, TD-60-depleted cells are apparently able to exit mitosis with normal, or even slightly elevated, kinetics.

This result was confirmed by live-cell imaging in U2OS cell lines, which revealed a modest, but statistically significant, delay of anaphase onset in TD-60-depleted cells (Supplementary Fig. 3b–d). Closer examination of fixed cells revealed that the delay in early mitosis correlated with a significant increase in the proportion of prometaphase cells with early prometaphase-like abnormal spindle shapes (Fig. 2c,d and Supplementary Figs 3e and 4a).

TD-60-depleted cells also exhibited an increased level of Aurora B staining at centromeres relative to control cells. Staining was scored for each centromere relative to the level of ACA staining for kinetochore proteins (Fig. 2e,f and Supplementary Fig. 4b). The increase in Aurora B levels at centromeres following TD-60 depletion was statistically significant (Fig. 2f). Survivin and INCENP levels also increased at centromeres following TD-60 depletion (Supplementary Fig. 4c); however, the levels of total histone H3 Serine 10 phosphorylation (H3S10ph) and H3 Serine 28 phosphorylation (H3S28ph) were not significantly altered (Supplementary Fig. 4e).

Given the functional connection observed between TD-60 and RalA *in vitro*, we also asked whether TD-60 depletion altered the subcellular distribution of RalA. The distribution of RalA in cells was typically diffuse/punctate, although in many cells the protein was concentrated near the plasma membrane and in telophase cells, some RalA was also observed in the intercellular bridge (Supplementary Fig. 4d). TD-60 was not required for RalA localization at the plasma membrane or in the cytoplasm consistent with the view that RalA is regulated by other GEFs at these sites^37^.

We conclude that TD-60 function in early mitosis is important for spindle assembly and regulation of the levels of the CPC at centromeres.

### RalA depletion perturbs spindles and centromeric Aurora B

RalA regulates vesicle trafficking, filopodia formation and cytoskeleton remodelling^24^. More recently, RalA has been shown to be required for segregation of the mitochondrial network during mitosis in a pathway that requires both cyclin B–Cdk1 and Aurora A kinase activity^38^.

**Figure 3:**
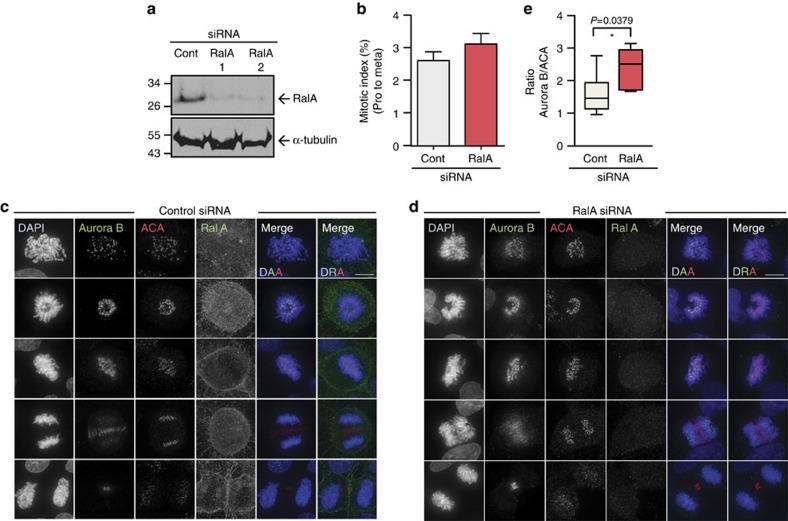
RalA ablation perturbs prometaphase spindles and centromeric Aurora B. (**a**) HeLa cells were transfected with siRNAs targeting two distinct regions of human RalA mRNA. Immunoblots of whole-cell extract (WCE) show RalA depletion after 72 h, with α-tubulin as loading control. (**b**) Mitotic index (prophase to metaphase) of RalA-depleted cells 72 h after transfection with siRNA (*n*=3). (**c**,**d**) Suppression of RalA with siRNA induces an increase of Aurora B level at centromeres. RalA-depleted cells were fixed with 4% PFA and stained with DAPI (blue), Aurora B (green), ACA (Cy5—red) and RalA (green). Scale bar, 10 μm. (**e**) Quantification of Aurora B intensity from the experiment illustrated in panel (**c**,**d**) using ImageJ and normalized. More information regarding quantification can be found in Supplementary Fig. 4b (*n*=15, *P<0.05 by Mann–Whitney *U*-test). DAPI, 4′,6-diamidino-2-phenylindole.

To further explore the functional interactions between TD-60 and RalA, we examined the mitotic phenotype following the suppression of RalA expression in HeLa cells. Immunoblotting showed that RalA was significantly depleted in asynchronous HeLa cultures treated with RalA-specific siRNAs (Fig. 3a)^39^. As was seen following TD-60 suppression, a modest increase in the percentage of cells in early mitosis was seen after 72 h of exposure to RalA siRNA (Fig. 3b). Indirect immunofluorescence confirmed the effectiveness of the RNAi, as cells transfected with RalA siRNA lacked detectable RalA at the plasma membrane and in the cytoplasm during mitosis (Fig. 3c,d).

RalA depletion resulted in phenotypes similar to those seen following depletion of TD-60. We observed both an increase in early prometaphase-like and abnormal mitotic spindle shapes resembling those seen following TD-60 depletion (Supplementary Fig. 5) and an accumulation of Aurora B at centromeres (Fig. 3c,d). The increase in centromeric levels of Aurora B relative to ACA after RalA ablation was statistically significant (Fig. 3e).

Together these results suggest that TD-60 and RalA might act in a common pathway to control spindle assembly and CPC distribution in mitosis.

### Loss of TD-60 or RalA raises prometaphase Kt-fibre stability

**Figure 4:**
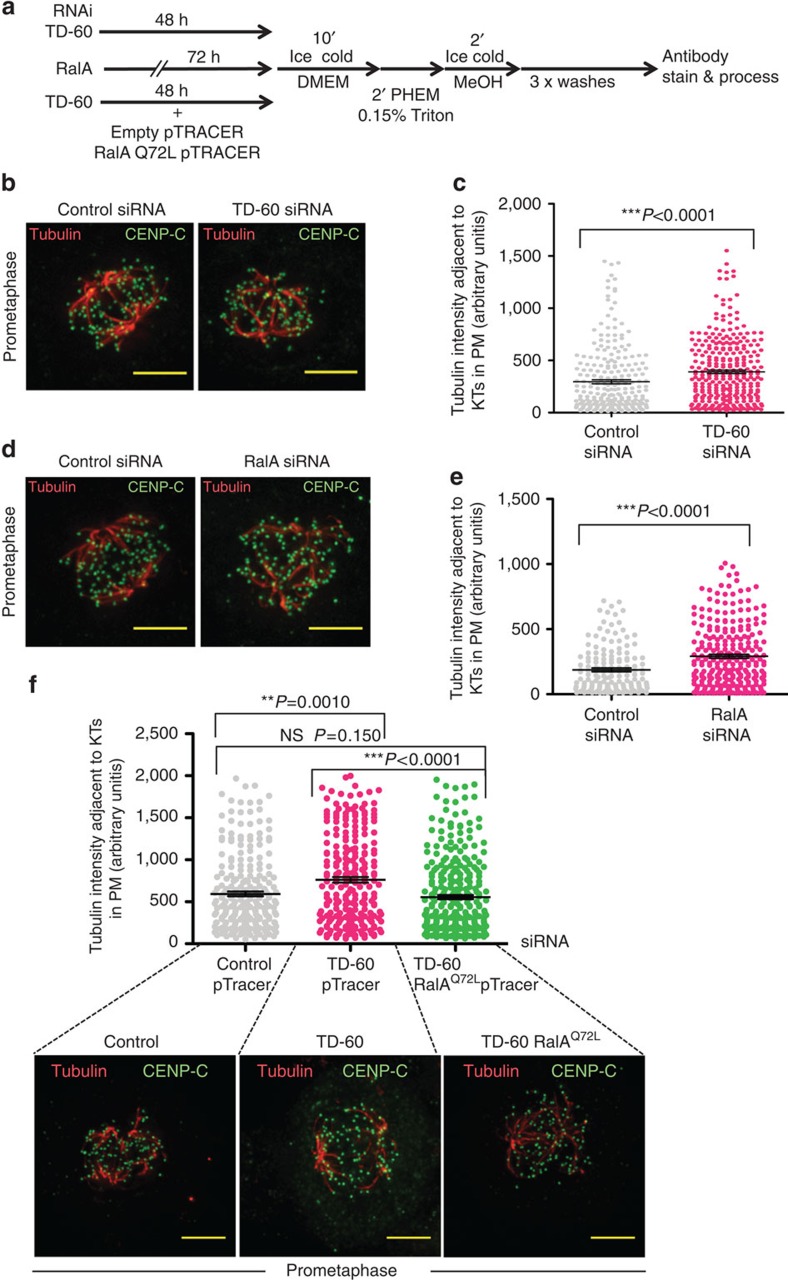
Increased Kt-MT density in TD-60 or RalA-depleted prometaphase cells is reversed by active RalA. (**a**) Protocol for experiment determining effect of TD-60 and RalA depletion and rescue experiments with RalAQ72L pTRACER on stability of kinetochore–microtubule binding. (pTRACER expresses an unlinked GFP that can be used to identify transfected cells expressing untagged protein.) (**b**) MT density (tubulin intensity) adjacent to kinetochores (KTs) in TD-60-depleted prometaphase cells. Control and TD-60-depleted cells were stained for CENP-C (green) and α-tubulin (red). Scale bar, 10 μm. (**c**) Quantification of the experiment in **b** using a kinetochore-recognition macro in ImageJ (*n*=5 cells from two independent experiments, ***P<0.0001 by Mann–Whitney *U*-test). (**d**) MT density (tubulin intensity) adjacent to kinetochores in RalA-depleted prometaphase cells. RalA-depleted cells were stained for CENP-C (green) and α-tubulin (red). Bar, 10 μm. (**e**) Quantification of the experiment in **d** using a kinetochore-recognition macro in ImageJ (from five cells each from two independent experiments). (**f**) High MT density (tubulin intensity) adjacent to kinetochores (KTs) observed in TD-60-depleted prometaphase cells, is restored in HeLa cells expressing RalAQ72L (GTP-locked). Control, TD-60-depleted and TD-60-depleted cells expressing RalAQ72L were stained for CENP-C (green) and α-tubulin (red). Scale bar, 10 μm. Quantification of the experiment was carried out using ImageJ (from five cells in each condition). The graph reports mean±s.e.m. (**P<0.005, ***P<0.0001 by Mann–Whitney *U*-test).

The abnormal spindle phenotype observed in TD-60 and RalA-depleted cells suggested that kinetochore–microtubule interactions might be altered in those cells. To test this hypothesis, cells transfected with TD-60 or RalA-specific siRNAs were cold treated and Kt-fibres (microtubule bundles attached to kinetochores) in prometaphase visualized by indirect immunofluorescence. Kinetochores were localized by CENP-C staining, and we then measured the tubulin staining intensity adjacent to them using a custom kinetochore-recognition macro in ImageJ^40^. A modest but statistically significant increase in kinetochore-associated cold-stable tubulin was observed following either TD-60 or RalA depletion (Fig. 4a–e)

We conclude that both TD-60 and RalA are required for prometaphase kinetochores to make normal and cold-stable attachments to microtubules.

### RalA Q72L rescues mitotic defects caused by TD-60 depletion

The similar phenotypes observed following the knockdown of both TD-60 and RalA led us to hypothesize that TD-60 activation of RalA might be required to regulate kinetochore–microtubule interactions in early mitosis. To test this hypothesis, we asked whether a constitutively active RalA mutant (RalA Q72L—the GTP-locked form) could rescue the phenotypes observed following transfection of HeLa cells with TD-60 siRNAs.

**Figure 5:**
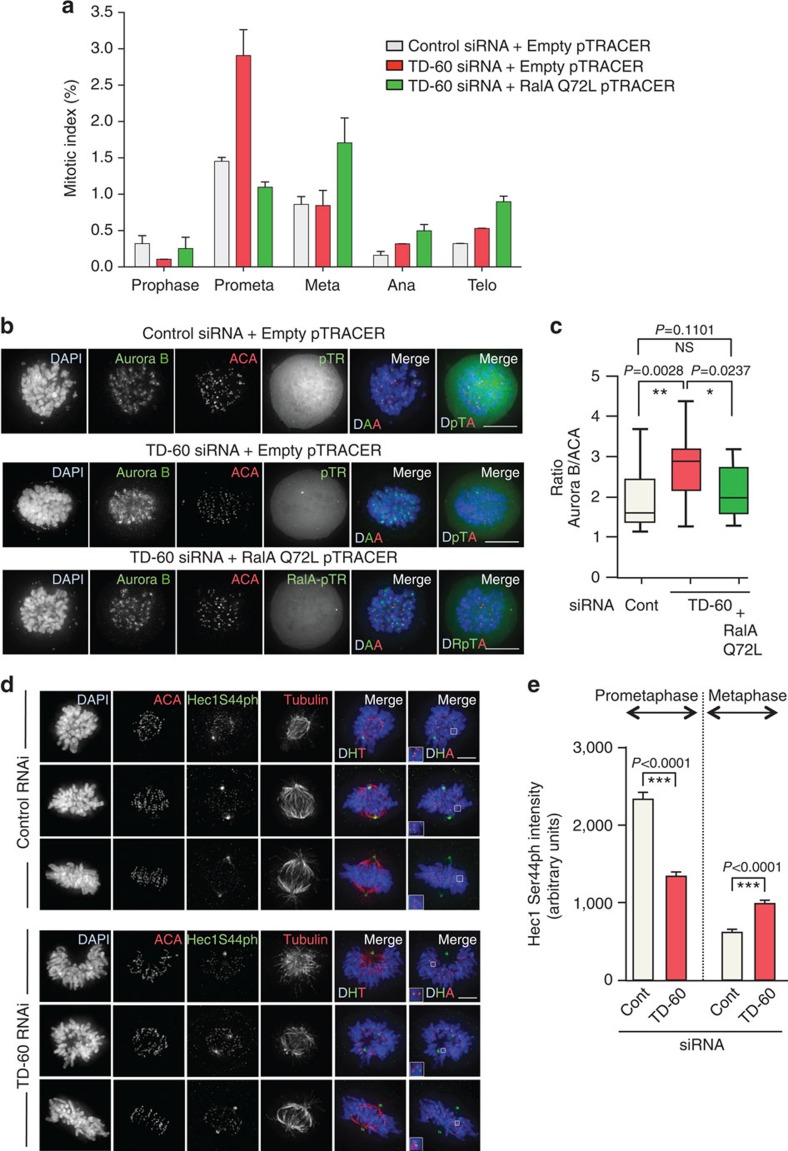
RalAQ72L mutant restores WT Aurora B levels at centromeres. (**a**) HeLa cells were co-transfected with TD-60 siRNA plus RalAQ72L mutant in pTRACER or Empty pTRACER, fixed with 4% PFA and stained for immunofluorescence and scoring. Graph reports the percentage of total cells in each mitotic phase (mean±s.e.m., *n*=2). (**b**) Immunofluorescence of HeLa cells co-transfected with Control siRNA and Empty pTracer (pTR), TD-60 siRNA and Empty pTracer or TD-60 siRNA and RalAQ72L pTracer (RalA-pTR), fixed with 4% PFA and stained for DAPI (blue), ACA (red) and Aurora B (green). Scale bar, 10 μm. (**c**) Quantification of the experiment in **b** using ImageJ. Aurora B intensity was normalized relative to ACA staining (*n*=18). More information regarding quantification can be found in Supplementary Fig. 4b. (**d**) TD-60 depletion affects Hec1 phosphorylation. HeLa cells transfected with TD-60 siRNA were stained with DAPI (blue), ACA (Cy5—red), α-tubulin (red) and pHec1 Ser44 (green). Scale bar, 5 μm. (**e**) ImageJ quantification of phospho-Hec1 staining from the experiment shown in **d** (*n*≅150 from 5 cells, *P<0.05, **P<0.005 and ***P<0.0001 by Mann–Whitney *U*-test). DAPI, 4′,6-diamidino-2-phenylindole.

Interestingly, expression of RalA Q72L in cells transfected with TD-60 siRNA restored WT Kt-fibre MT density in prometaphase HeLa cells as determined using the cold-stable microtubule assay (Fig. 4f), and appeared to rescue the prometaphase delay (Fig. 5a) and the excess accumulation of Aurora B at centromeres normally observed following TD-60 depletion (Fig. 5b,c). These experiments further confirm a functional interaction between TD-60 and RalA *in vivo*.

### TD-60 depletion reduces Aurora B activity in prometaphase

As shown here, prometaphase cells depleted of TD-60 or RalA exhibit both increased levels of centromere-associated CPC and microtubule density at kinetochores. These two observations could be reconciled if the phosphorylation of Hec1/Ndc80 on Ser44 by Aurora B, which regulates the affinity of the kinetochore for microtubules, was perturbed in TD-60-depleted cells^41^,^42^,^43^. Indeed, we did observe a statistically significant decrease in the levels of Hec1 phosphorylation in prometaphase cells, while there was a slight increase in metaphase cells (Fig. 5d,e). This would be expected to stabilize microtubule attachments in prometaphase and therefore perhaps to slow the recovery of TD-60 and RalA-depleted cells from a block with the Eg5 inhibitor monastrol^44^. Indeed, although TD-60 or RalA-depleted cells could recover from such a block and form normal bipolar spindles, the process did appear to be slightly delayed (Supplementary Fig. 6).

**Figure 6:**
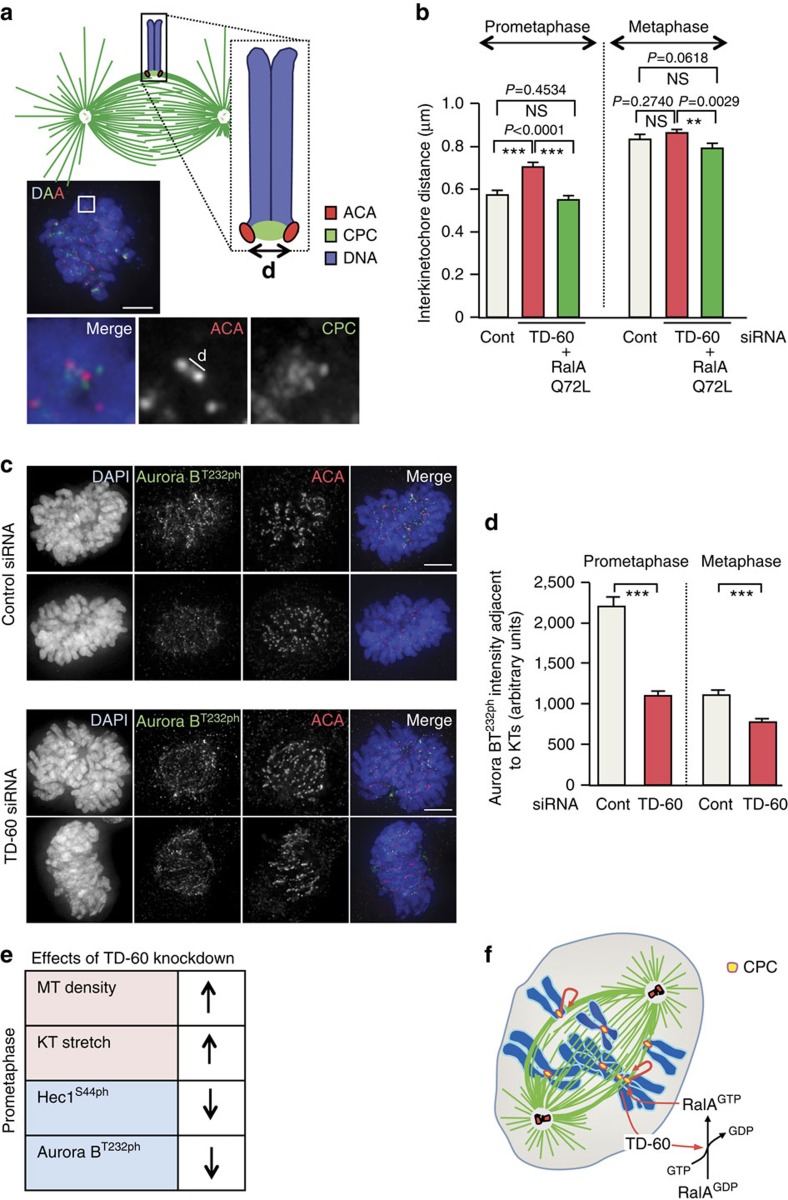
TD-60 depletion affects prometaphase Aurora B activity and kinetochore stretch. (**a**) Schematic representation of kinetochore stretch. Immunofluorescence of representative HeLa cell transfected with control siRNA and empty pTracer showing method used for measurement of stretching. HeLa cells were fixed with 4% PFA and stained for DAPI (blue), ACA (red) and Aurora B (green). Scale bar, 5 μm. (**b**) HeLa cells co-transfected with TD-60 siRNA±RalA Q72LpTRACER (shown in Fig. 5b) were stained for DAPI, ACA and Aurora B and the inter-kinetochore distance (μm) was determined using ImageJ. The graph reports mean±s.e.m. (**P<0.005, ***P<0.0001 by Mann–Whitney *U*-test). (**c**) HeLa cells transfected with Control or TD-60 siRNA were stained for DAPI (blue), ACA (red) and Aurora B-T232ph (T-loop) (green). Scale bar, 5 μm. (**d**) ImageJ quantification of centromeric phospho-Aurora B staining using a custom kinetochore-recognition macro in ImageJ^40^ from the experiment shown in (**c**) (*n*≅200, from 5 cells). The graph reports mean±s.e.m. (***P<0.0001 by Mann–Whitney *U*-test). (**e**) Table summarizing phenotypes observed after TD-60 depletion. (**f**) Cartoon illustrating how TD-60 activation of RalA could regulate Aurora B distribution at centromeres. Microtubules are in green, DNA in blue and the CPC in yellow. DAPI, 4′,6-diamidino-2-phenylindole.

A decrease in Hec1Ser44ph at kinetochores would be expected to be associated with more robust kinetochore–microtubule attachments, as conventionally detected in ‘kinetochore stretch' assays (Fig. 6a). We therefore measured the kinetochore stretch of prometaphase and metaphase chromosomes in control and TD-60-depleted cells. Consistent with the MT density and Hec1S44ph measurements, we observed a greater inter-kinetochore stretch in prometaphase cells depleted of TD-60 (Fig. 6b). Importantly, this increased tension at prometaphase kinetochores is apparently due to the loss of TD-60's GEF activity for RalA, since the expression of RalA Q72L in TD-60-depleted cells restored the sister-kinetochore spacing to WT values (Fig. 6b).

It was not initially obvious how the increase in Aurora B levels at centromeres seen after TD-60 or RalA depletion would result in the apparent decrease in Aurora B activity at prometaphase kinetochores observed in the Hec1 phosphorylation and kinetochore stretch assays. We therefore measured a direct marker of Aurora B activity status—autophosphorylation of Thr232 in the ‘activation loop' of the kinase—using a validated phospho-specific antibody^45^. Quantification of Aurora B-T232ph levels adjacent to ACA (using a custom kinetochore-recognition macro in ImageJ^40^) revealed that centromeric Aurora B appeared to be significantly less active in prometaphase (Fig. 6c,d). The levels of active kinase were also diminished in metaphase, though to a lesser degree.

As a control for the experiments in which RalA Q72L was shown to reverse the effects of TD-60 depletion, we expressed both RalA WT and RalA Q72L in HeLa cells and measured the inter-kinetochore distance in both prometaphase and metaphase cells. Minimal differences were seen when we compared transfected and untransfected cells, with a slight decrease seen in prometaphase cells expressing RalAQ72L and a slight increase seen in metaphase cells expressing RalA WT (Supplementary Fig. 7). In all cases, the changes were far less than those observed in the RNAi-rescue experiments.

As summarized in Fig. 6e, these results suggest that TD-60-associated RalA GEF activity contributes to regulation of kinetochore–microtubule stability in prometaphase, both by regulating CPC levels and activity at centromeres.

## Discussion

The reported localization of TD-60 to centromeres, the spindle midzone (the ‘Telophase Disk') and midbody resembles that of the CPC^1^,^2^ and TD-60 has been proposed to be either a component of the CPC or functionally closely associated with it^5^,^10^,^11^. The experiments reported here reveal that TD-60, through its action as a GEF for RalA, does influence the localization and activity of the CPC at centromeres during early mitosis. Thus, TD-60 apparently functions in conjunction with the CPC, but not as a part of the complex itself.

Other studies have found links between TD-60/RCC2 and cell migration, membrane trafficking and cancer cell invasiveness^14^,^16^,^17^. These roles for the protein appeared at odds with its presumed mitotic functions, but now fit very well with our discovery that TD-60 is a bona fide GEF for RalA. RalA is involved in membrane trafficking, regulation of cell shape and movement, cytokinesis and anchorage-independent growth of transformed cells^21^,^24^. RalA effectors that bind activated RalA include RalBP1 and the exocyst^46^. RalA also interacts in a GTP-independent manner with calmodulin and is involved, with Arf6, in the activation of phospholipase D^21^. In these non-mitotic roles, TD-60 and RalA appear to share a number of interacting partners and functional roles. It is noteworthy, given the links between RalA and cancer, that the TD-60/RCC2 gene shows reduced copy number in >33% of breast, colon, lung and ovarian cancers catalogued in the COSMIC database^47^. Furthermore, that database shows that several residues located on the predicted RalA-binding surface of TD-60 can undergo missense mutations in cancer.

We find that TD-60 has GEF activity for RalA, but not for RalB (or Rac1 or Ran). This distinction is not unexpected. RalA and RalB share 80% sequence identity, but whereas RalA is dispensable for life in HeLa cells, RalB is essential^22^. Furthermore, RalA and RalB function at distinct stages of cytokinesis and may be activated by different GEFs^48^. Moreover, electrostatic surface potential calculations also highlight noticeable differences between RalA and RalB in their putative TD-60-binding surfaces (Supplementary Fig. 8a). A closer look at the amino-acid composition of their TD-60-binding interfaces also shows the presence of at least three variations (Supplementary Fig. 8b) that might explain the RalA-specific activity shown by TD-60.

The role of RalA in mitotic regulation has been less widely studied than those of Rac, RhoA and Cdc42. Together with the exocyst, RalA has a role in cytokinesis, although the exact details of this are disputed^39^,^48^. More recent studies have revealed that RalA activated by Aurora A kinase phosphorylation^23^,^49^ relocates to mitochondrial membranes in metaphase^24^. There it acts together with cyclin B/Cdk1 to regulate mitochondrial fission via targeting of the GTPase Drp1 (refs 38, 50).

Our studies reveal that TD-60 and RalA—likely acting at least in part via the CPC—modulate kinetochore–microtubule interactions during mitosis (Fig. 6f). Perhaps surprisingly, despite our data clearly revealing a number of functional connections between RalA activation and kinetochore function, antibody staining did not reveal a concentration of RalA near centromeres. We propose that RalA may function in mitosis like Ran—by forming gradients of activity near its sites of action that are created through the localization of its relevant GEF^51^,^52^. Thus, much as Ran is activated in a gradient close to mitotic chromosomes where its GEF RCC1 binds^53^,^54^, RalA may be activated near centromeres as a result of the localization of TD-60. It will be interesting in future experiments to map the distribution of active RalA in mitotic cells, as has been done for Ran, though this is likely to be complicated, given that there are at least six other GEFs for RalA.

A major function of the CPC during prometaphase is to regulate the microtubule binding affinity of the kinetochore^55^,^56^. It does this by phosphorylating outer kinetochore proteins such as Ndc80/Hec1 and the Knl1 complex^41^,^42^,^57^,^58^. This reduces their affinity for microtubules and promotes microtubule release, thereby allowing error correction. Our results now add TD-60/RalA as an additional control input to this important process.

In prometaphase, TD-60/RalA appears to promote dynamic interactions between kinetochores and microtubules. Thus, on TD-60 depletion, spindle tension (measured by sister-kinetochore stretch) and Kt-MT density are increased, recovery from a monastrol block is delayed, and prometaphase spindle structure is abnormal. These effects apparently depend on the GEF activity of TD-60 for RalA, since they can be reversed by the expression of RalA Q72L, and may at least be partly explained by a decrease in the phosphorylation of Hec1Ser44 by Aurora B. The continued ability of TD-60/RalA-depleted cells to form Kt-MT attachments might explain why these cells are, after a delay, apparently able to satisfy the mitotic checkpoint and exit mitosis.

These results are particularly interesting in view of a recent study that described a significant difference in the dynamics of kinetochore–microtubule interactions between prometaphase and metaphase^59^. The authors argued that this difference (more dynamic interactions in prometaphase versus more static interactions in metaphase) was due to the activity of Cdk1–cyclin A, with the prometaphase/metaphase switch requiring destruction of cyclin A by the proteasome. Considering their results together with ours, it is interesting to speculate that Cdk1–cyclin A might directly or indirectly activate the relevant GEF activity of TD-60. Alternatively, RalA-GTP might function in a signalling pathway alongside Cdk1–cyclin A, much as it does with Cdk1–cyclin B in mitochondrial segregation.

This study provides the first example of a functional interaction between a small GTPase and the CPC in mitotic regulation. The mechanisms by which RalA regulates CPC level and activity at the centromere remain to be determined, and in the future it will be important to determine whether RalA impacts on the various systems that target the CPC to centromeres^60^,^61^,^62^ or on systems that remove the CPC from chromatin^63^,^64^, as well as on CPC-independent regulatory mechanisms in mitosis and beyond.

## Methods

### Cell culture

HeLa Kyoto (EMBL Heidelberg, Germany), U2OS EGFP-γ-tubulin/mRFP-H2B^65^ and U2OS GFP-α-tubulin cells (a gift from Duane Compton, Dartmouth, USA) were grown in Dulbecco's modified Eagle's Medium supplemented with 5% (v/v) FBS (fetal bovine serum) and 100 U ml^−1^ penicillin–streptomycin, at 37 °C and 5% CO_2_ in a humidified incubator.

### Plasmids

Human SBP-TD-60 was codon optimized, synthetically generated and subcloned in pFastBacI by GeneArt. The human RalA Q72L mutant was generated by site-directed mutagenesis using (FW 5′-agatacagctgggctggaggactacgctg-3′; RV 5′-cagcgtagtcctccagcccagctgtatct-3′) and RalA G26A mutant was generated by site-directed mutagenesis using (FW 5′-agtggtggcgtggccaagtcagctctg-3′; RV 5′-cagagctgacttggccacgccaccact-3′). RalA WT was used as a DNA template. Mutants generation was performed according to standard protocols using a Stratagene QuickChange II XL. RalA Q72L was cloned into pTRACER_SV40 (Life Technologies). For pull-down assays, human RalA WT, G26A and Q72L were cloned into pEGFP-C2 (Clontech). For far-western assay human RalA and Ran were cloned into pFAT2.

### Antibodies

For immunofluorescence analysis, we used the following primary antibodies: anti-Aurora B rabbit polyclonal antibody (Abcam ab2254, 1:1,000), anti-AIM-1 mouse monoclonal antibody (BD Bioscience 611,082, 1:100), anti-Tubulin B512 mouse monoclonal antibody (Sigma T5168, 1:1,000), anti-INCENP P240 rabbit polyclonal antibody (Cell Signalling 2,807, 1:400), anti-Survivin rabbit polyclonal antibody (Novus Biological NB500-201, 1:200), anti-H3S10ph rabbit polyclonal antibody (Merck Millipore 06-570, 1:500), anti-H3S28ph rabbit polyclonal antibody (Cell Signalling 9,713, 1:200), anti-RalA mouse monoclonal antibody (BD Bioscience 611,022, 1:50), anti-Hec1S44ph rabbit polyclonal antibody (kindly supplied by Dr Jennifer DeLuca^43^), anti-CENP-C rabbit polyclonal antibody^66^, anti-Aurora B-T232ph rabbit polyclonal antibody^45^ and anti-ACA human serum^67^. For secondary incubations, we used antibodies conjugated with Alexa (Life Technologies) Fluor 594 (rabbit a21207 and mouse a21203, 1:1,000), Fluor 488 (rabbit a21206 and mouse a21202, 1:500) and Flour 647 (rabbit a31573 and mouse a31571, 1:250) and anti-human Alexa Fluor 647 (Jackson Immunoresearch 709-605-149, 1:250 )

### RNA interference

For both TD-60 and RalA depletion, 0.8 × 10^5^cells ml^−1^ of a logarithmically growing HeLa culture were transfected with target specific siRNA duplexes^39^, using HiPerfect transfection reagent (QIAGEN) according to the manufacturer's protocol. A control siRNA (QIAGEN) that shows no homology to any known mammalian gene was used as a negative control.

HeLa cells were transfected with 150 nM of TD-60 siRNAs covering two separate regions (siRNA_1:_1500._aagagatgaaagtgagactga_1520_; siRNA_2: _535_aaggggcagctgggacatggt_555_) for 48 h (ref. 10).

HeLa cells were transfected with 50 nM of RalA siRNAs covering two separate regions (siRNA_1: _17_ ccaagggtcagaattcttt_36_; siRNA_2: _484_ gctaatgttgacaaggtat_503_ ) for 72 h (ref. 39).

For TD-60 siRNA rescue experiments, HeLa cells were co-transfected with 150 nM of TD-60 siRNA (siRNA_2) plus 6 μg DNA encoding RalA Q72LpTRACER using PolyPlus transfection reagent (JetPRIME) according to the manufacturer's protocol.

### Indirect immunofluorescence

HeLa cells were grown on polylysine-coated coverslips (Poly-L-lysine solution—SIGMA) and fixed respectively with paraformaldehyde (PFA) or ice-cold methanol (MeOH). For PFA fixation, cells were fixed for 10 min with 4% (v/v) paraformaldehyde (Electron Microscopy Services) in warm 1 × buffer PBS. Cells were permeabilized with 0.15% Triton-X-100 in 1 × PBS for 2 min at room temperature. After permeabilization, coverslips were incubated with 1% (v/v) bovine serum albumin/PBS blocking solution for an hour at room temperature. For MeOH fixation, cells were fixed for 10 min with ice-cold methanol and then washed in PBS 1 × . Fixed cells were then stained with specific antibodies.

### Cold-stable microtubule assay

Transfected cells were incubated with ice-cold Dulbecco's modified Eagle's Medium for 10 min, followed by 2 min with PHEM buffer pH 6.9 (60 mM PIPES, 25 mM HEPES, 10 mM EGTA, 2 mM MgCl_2_), and fixed in ice-cold MeOH for 2 min. Cells were stained with specific antibodies as described above.

### Imaging

Three-dimensional data sets were acquired using a CoolSnap HQ cooled CCD camera (Photometrics), on a DeltaVision Spectris microscope (Applied Precision, LLC, WA) with a × 100/1.4NA PlanApo lens. Optical sections were acquired every 0.2 μm, and three-dimensional data sets were deconvolved using the constrained iterative algorithm^68^,^69^.

Live-cell imaging were conducted using a Nikon Ti-E inverted microscope with a × 20/0.75 NA objective. Images were acquired using Nikon DS Qi1MC camera with a resolution of 1,024 × 1,024 pixels, every 5 min for 22 h in a 5% CO_2_/37 °C chamber.

Cold-stable microtubules, Hec1S44ph and Aurora B-T232ph were quantified using a macro based on the default setting for CraQcode v1.06 (ref. 40) as modified in the Earnshaw lab by Nuno Martins. This macro uses ACA to detect kinetochores, and then draws a box around the centromere to define a region for quantification of the data channel. The Aurora B level in TD-60 and RalA siRNA experiments was quantified using ImageJ, using ACA (Cy5) as a reference. Intensity values of ∼20 cells were exported in Excel, which was also used to calculate the Aurora B/ACA ratio (see Supplementary Fig. 4b). Inter-kinetochore distances between sister kinetochores as detected by ACA staining were measured in ImageJ. Classification of mitotic stages and spindle phenotypes was carried out by a blinded observer. For statistical analysis and graphic representation, we used Prism 5 (Graphpad). For statistical analysis of the data, we used an unpaired Student's *t*-test (***P<0.0001) or Mann–Whitney *U*-test (*P<0.05; **P<0.005; ***P<0.0001).

### Immunoblotting

Whole-cell extracts were prepared from HeLa cells transfected with control siRNA, TD-60 siRNA or RalA siRNA. Immunoblotting analysis was performed using the following primary antibodies: anti-RCC2 rabbit polyclonal antibody (Cell Signalling 1:100–1:200), anti-RalA mouse monoclonal antibody (BD Bioscience 1:100), anti-Ran mouse monoclonal antibody (BD Bioscience 1:1,000), anti-Tubulin B512 mouse monoclonal antibody (Sigma 1:10,000), anti-Beta Actin mouse monoclonal antibody (Sigma 1:1,000) and anti-GFP rabbit polyclonal (Life Technology, 1 μg ml^−1^). For protein detection and quantitation, we used donkey IRDye 800 secondary antibodies (LI-COR Bioscience 1:10,000), HRP-conjugated anti-mouse secondary antibody (GE Amersham 1:5,000) and anti-rabbit IgG, HRP-linked Antibody (Cell Signalling 1:3,000). Uncropped versions of western blots can be found in Supplementary Fig. 9.

### GFP pull-down assay

HeLa Kyoto cells were grown in 10-cm dishes and transfected with RalA WT, G26A and Q72L in pEGFP-C2 and then incubated for 48 h at 37 °C. pEGFP-C2 empty plasmid was used as a GFP control. GFP pull-down assays were performed by using GFP-Trap-M (GFP-binding) magnetic beads (Chromotek) according to the manufacturer's protocol. The lysis buffer used was 50 mM Tris pH 8.0, 0.4 M NaCl, 0.5% NP-40, 0.5% deoxycholate, 1:1,000 protease inhibitor cocktail P-8340 (Sigma) and 100 μM PMSF. The dilution buffer was 50 mM Tris pH 8.0, 0.4 M NaCl, 0.1% deoxycholate, 1:1,000 protease inhibitor cocktail P-8340 (Sigma) and 100 μM PMSF. Immunoprecipitation samples were analysed by immunoblotting using specific primary antibodies for GFP tag (Invitrogen) and TD-60/RCC2 (Cell Signalling). Anti-rabbit IgG, HRP-linked antibody (Cell Signalling) was used for ECL detection.

### GEF assay

For TD-60 GEF activity, we performed a GDP-release assay as previously described^30^,^70^. In brief, 10 μg of each pre-selected GST-tagged GTPase was added to the following mixture (125 μM EDTA, 50 mM HEPES-NaOH pH 6.8, 10 μM Mg-GDP, 0.1 mg ml^−1^ bovine serum albumin), plus 5 μCi [3H]-GDP (10 mCi ml^−1^; 5,000 Ci mmol^−1^) in a total volume of 200 μl. This mixture was incubated for 15 min at 30 °C to load the selected GTPases with the radioactive probe. After adding 20 μl of 10 mM Mg-GTP, the loading reaction was split into two tubes (110 μl each), and was then incubated, respectively, with 10 nM SBP-tagged recombinant TD-60 or a buffer control. After −/+GEF samples were incubated for 20 min at 30 °C, 2.5 μl was taken for a specific activity measurement. The remainder was split into two tubes and bound to 20 μl glutathione sepharose beads diluted in 500 μl of ice-cold GEF assay buffer for 1 h at 4 °C. After four washes with 500 μl of ice-cold GEF assay buffer, beads were transferred into 4 ml of scintillation fluid for counting. The total amount of nucleotide exchange was calculated as pmoles of GDP released.

### Cellular RalA activity assay

RalA-GTP levels were measured in HeLa cells using the RalA G-LISA kit (Cytoskeleton) according to the manufacturer's protocol. RalA-GTP was detected by using specific primary RalA antibody provided in the kit. Lysates were tested at a concentration of ∼0.5 mg ml^−1^.

## Additional information

**How to cite this article:** Papini, D. *et al.* TD-60 links RalA GTPase function to the CPC in mitosis. *Nat. Commun.* 6:7678 doi: 10.1038/ncomms8678 (2015).

## Supplementary Material

Supplementary Information Supplementary Figures 1-9, Supplementary Table 1 and Supplementary Methods
