# Supplementary material for: TD-60 links RalA GTPase function to the CPC in mitosis
Source: Nat Commun. 2015 Jul 9;6:7678. doi: 10.1038/ncomms8678 (PMC4510650; doi:10.1038/ncomms8678)
Supplement: Supplementary Information — Supplementary Figures 1-9, Supplementary Table 1 and Supplementary Methods [file ncomms8678-s1.pdf]

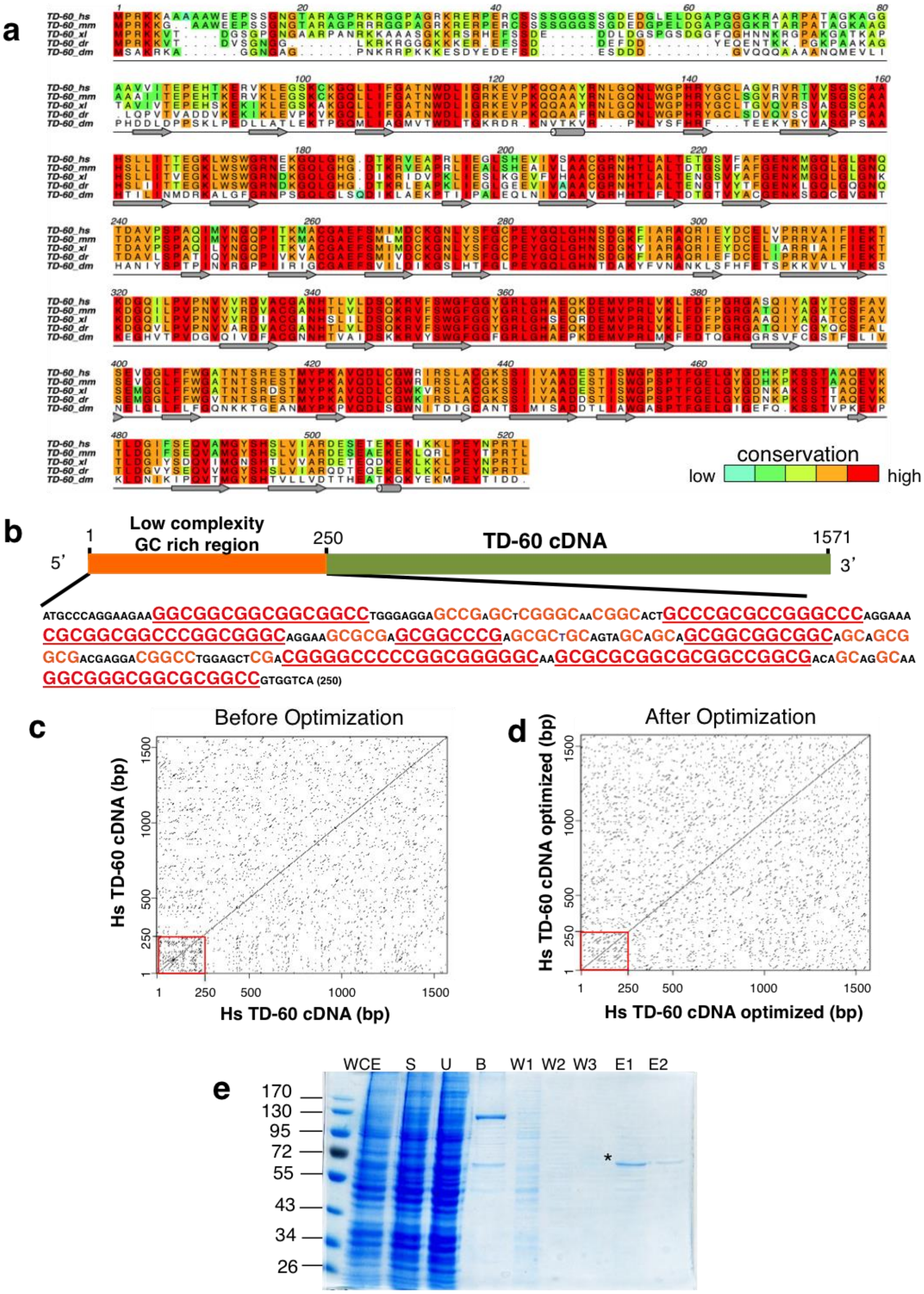

Supplementary Figure 1. Identification of a low-complexity GC rich near the 5' end of the TD-60 cDNA allowed its expression and purification.

(a) Multiple sequence alignment of TD-60 highlighting the amino acid conservation between *H. sapiens* (hs), *M. musculus* (mm), *X. laevis* (xl), *D. rerio* (dr) and *D. melanogaster* (dm). Predicted secondary structure elements are shown below the sequence alignment. Colour codes represent different degrees of amino acid conservation (Cyan, Blue, Yellow, Orange and Red colours represent 33%, 50%, 67%, 83% and 100% sequence conservation, respectively). Multiple sequence alignment was carried out using ClustalW and the figure was generated using Aline. **(b)** Schematic representation of human TD-60 cDNA before codon optimization. The low complexity GC-rich region at the 5' end of the gene is 250 bp in length. **(c)** Dotplot of human TD-60 cDNA showing the low complexity region at the 5' end. The graph was plotted in seqinR using a window of 10 bp, looking for matches of 6 or more, and stepped along the region in 4 bp intervals **(d)** Similar dotplot of human synthetic codon optimized TD-60 cDNA. **(e)** SBP tagged TD-60 was expressed in SF9 cells using a baculovirus vector. Human TD-60 was purified from large scale SF9 culture infected with high MOI TD-60 virus. Staining of purified recombinant TD-60 using InstantBlue reagent (Expedeon).

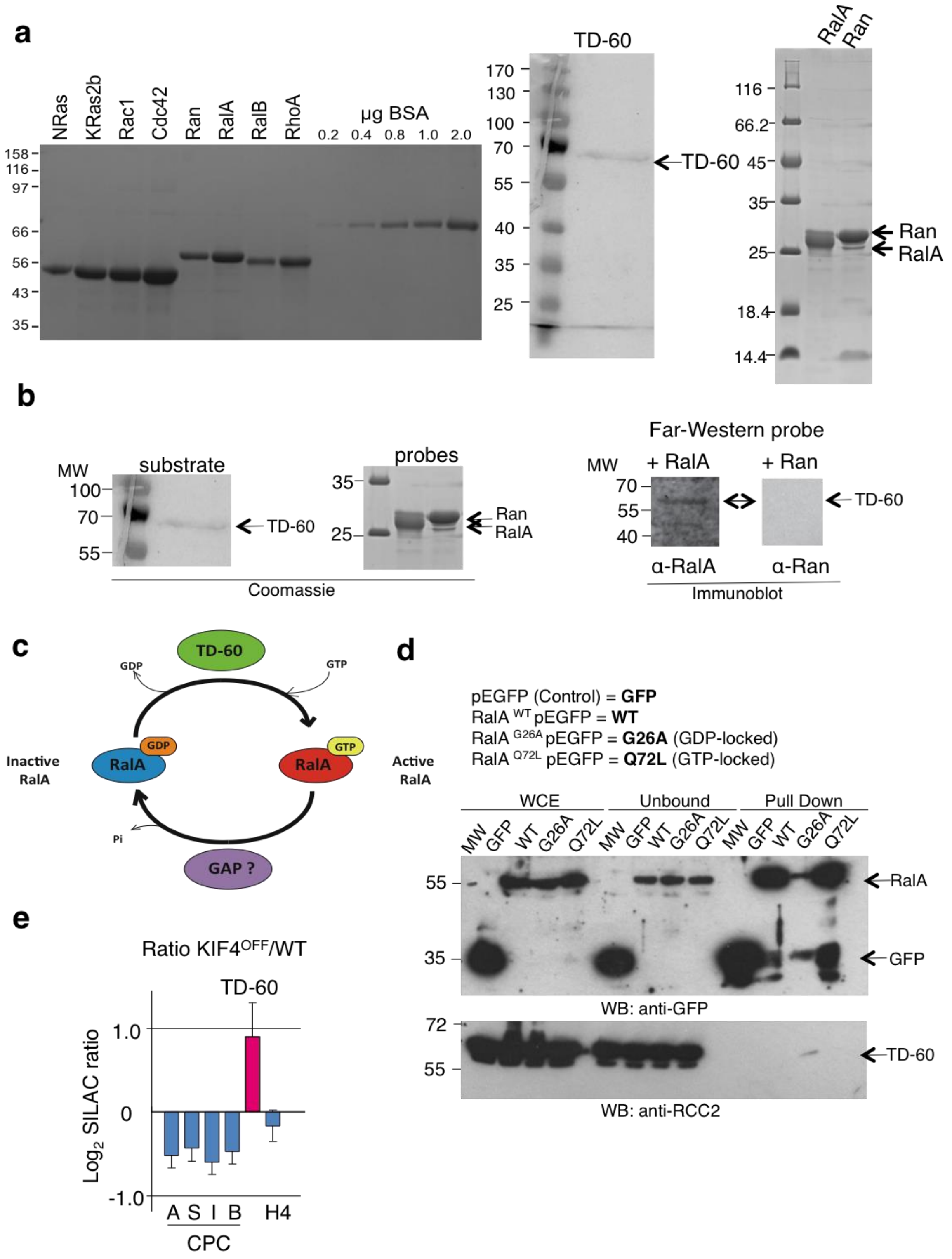

Supplementary Figure 2. Far-western and pull down assay of TD-60 and its GTPase target.
(a) Recombinant GST-GTPases for GEF assay were expressed in E.*coli*. Protein purity was tested and quantified by SDS-PAGE in comparison with a BSA standard. Gels showing the purity of other GTPases used are shown in[27, 28]. Staining of purified recombinant TD-60 and tagged cleaved RalA and Ran using InstantBlue reagent (Expedeon). **(b)** Far-western analysis reveals that recombinant SBP-TD-60 binds to recombinant RalA, but not Ran. **(c)** Schematic diagram of TD-60/RalA GTPase cycle. **(d)** Pull Down assay of RalA WT, G26A (GDP-locked), and Q72L (GTP-locked) GFP tagged from asynchronous HeLa cells. Independent replica experiment of TD-60 – RalA interaction (WB: anti-GFP and anti-RCC2; see also Figure 1f). RalA G26A (GDP-bound) mutant was slightly insoluble, therefore to obtain an equal loading on the SDS-PAGE gel, a larger amount of protein sample was run (≈3 times more than other samples). **(e)** H/L SILAC Ratio of KIF4^{OFF} / WT purified chromosomes from DT40 (Chicken cells) showing the protein levels of the CPC (A=Aurora B; S=Survivin; I=INCENP; B=Borealin), TD-60 and Histone 4 (H4). Graph reports standard error of the mean (SEM) (n=3).

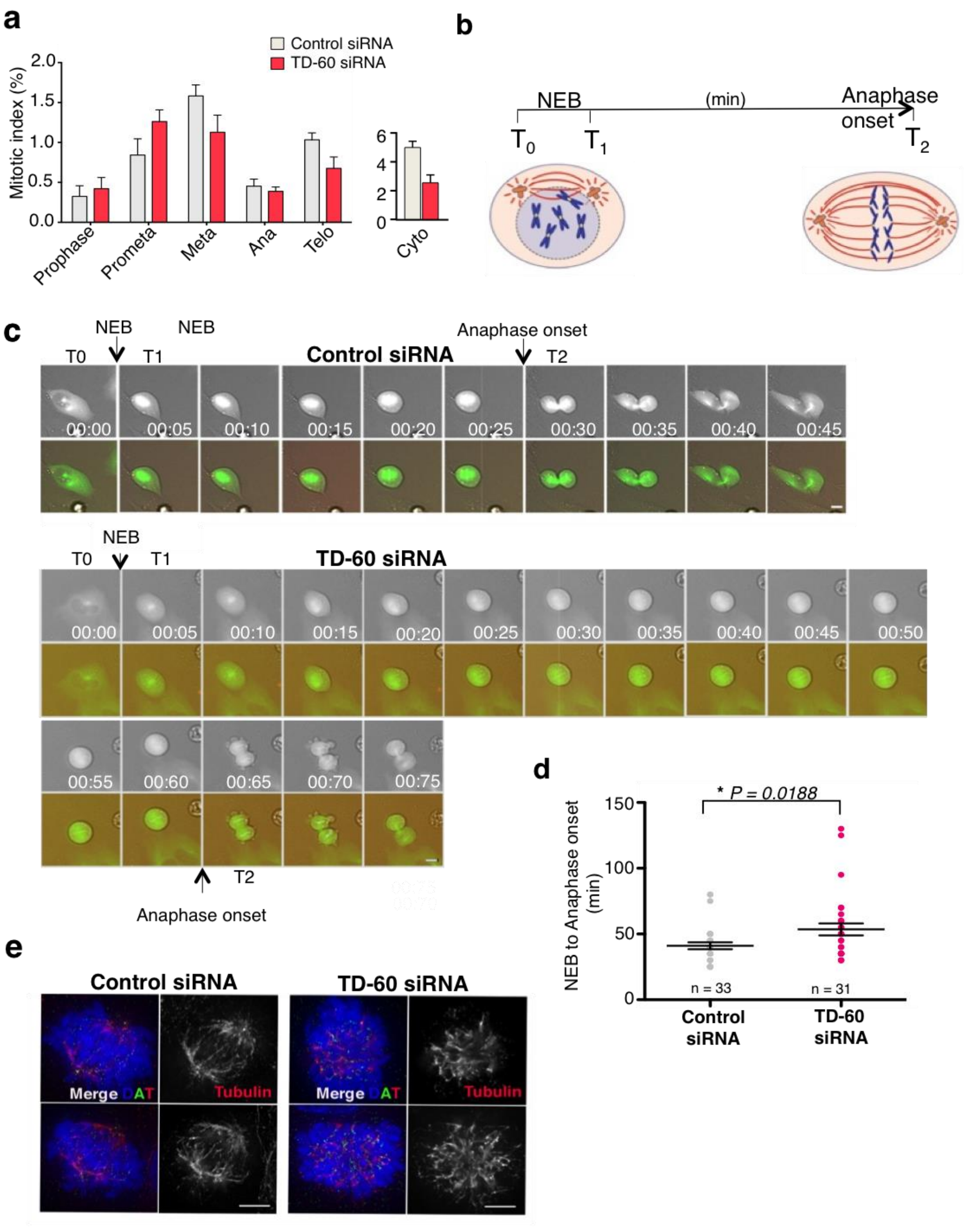

Supplementary Figure 3. Live cell imaging upon TD-60 siRNA in GFP-?-tubulin U2OS cells reveals a slight delay in anaphase onset.

(a) HeLa cells were co-transfected with TD-60 siRNA and fixed with 4% PFA and stained for immunofluorescence and scoring. Graph reports the mitotic index (%) of each mitotic phase (mean ± SEM, n=3). Cells in cytokinesis were scored separately. **(b)** Protocol for determining effect of TD-60 depletion on mitotic progression, between Nuclear Envelope Breakdown (NEB) (T1) and anaphase onset (T2). Cells were imaged every 5 minutes over 22 hours. **(c)** Representative images acquired for time points from NEB to anaphase onset of cells transfected with control or TD-60 siRNA. Bar, 10 µm. **(d)** Mitotic length quantification (min) between NEB and anaphase onset for GFP-?-tubulin U2OS cells. Graph reports mean ± SEM (n≈30). The Mann-Whitney test was used for statistical analysis. **(e)** High magnification of abnormal spindle shapes observed after TD-60 ablation. Control and TD-60-depleted cells fixed with 4% PFA were stained with DAPI (blue), α-tubulin (red) and ACA (green). Bar, 5 µm.

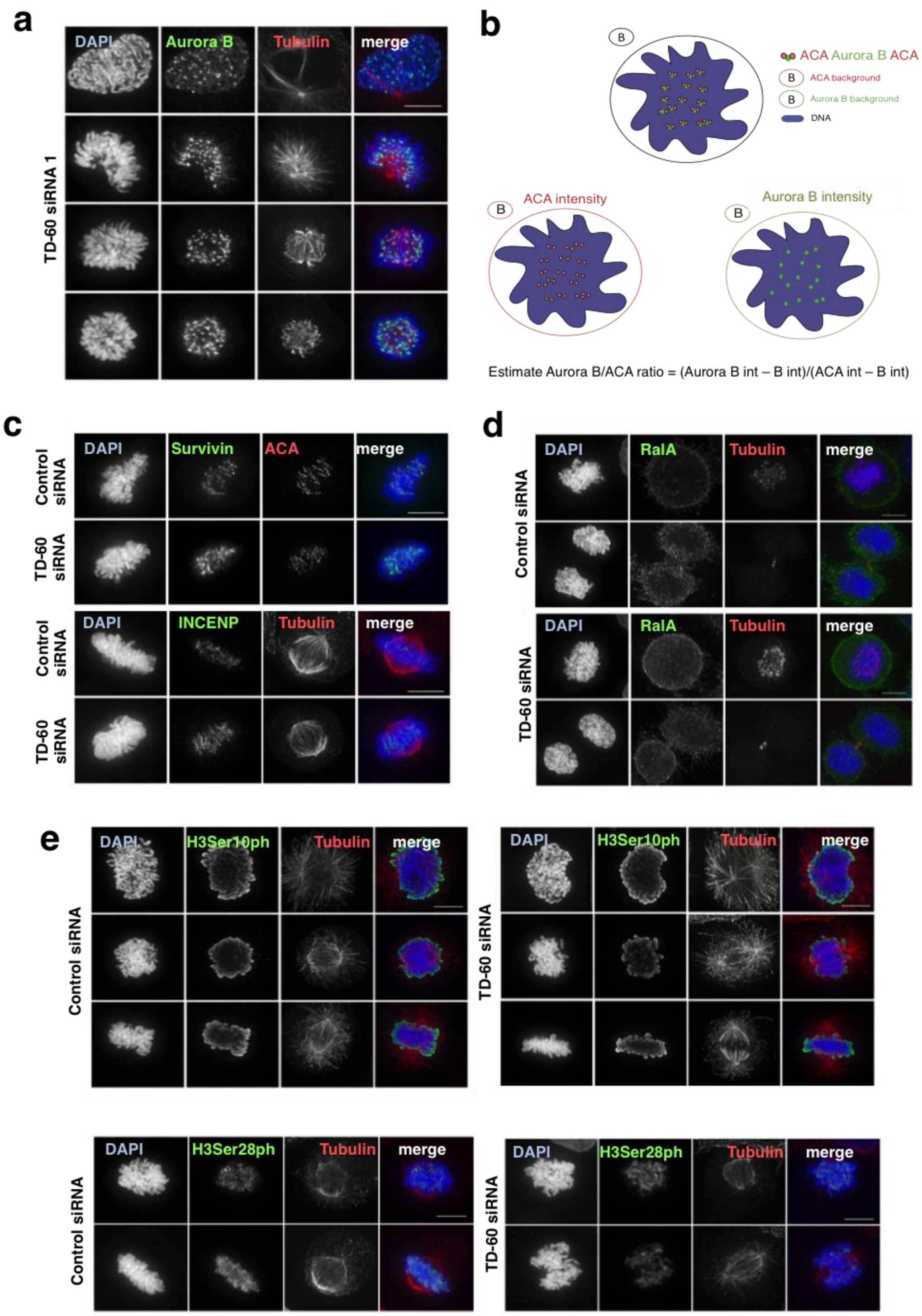

Supplementary Figure 4. TD-60-depletion affects levels of centromeric CPC.
(a) TD-60 knockdown phenotype is consistent using a second siRNA (TD-60 siRNA_1). TD-60 depleted cells were fixed with 4% PFA and stained with DAPI (blue), α-tubulin (red) and aurora B (green). Bar, 10 μm. **(b)** Schematic representation of method used for estimate quantitation of Aurora B intensity in early mitosis. Aurora B and ACA intensity was calculated by subtracting mean fluorescence intensity of a background region in the Aurora B and ACA channel (background or B) from the mean fluorescence intensity of total Aurora B or ACA in the chromatin region, which is shown in green or red respectively. The Aurora B/ACA ratio was calculated according to the formula shown in the figure. **(c)** The level of Survivin and INCENP at centromeres was increased in the absence of TD-60. TD-60-depleted cells were fixed with 4% PFA and stained with DAPI (blue), α-tubulin (red), ACA (Cy5), and Survivin or INCENP respectively (green). Bar, 10 μm. **(d)** The distribution of RalA is not altered following TD-60 RNAi. TD-60-depleted cells were fixed with 4% PFA and stained with DAPI (blue), Aurora B (red) and RalA (green). Bar, 5 μm. **(e)** Levels of H3S10ph and H3S28ph are not affected following TD-60 RNAi. TD-60-depleted cells were fixed with 4% PFA stained with DAPI (blue) α-tubulin (red), H3Ser10ph or H3Ser28ph respectively (green). Bar, 10 μm.

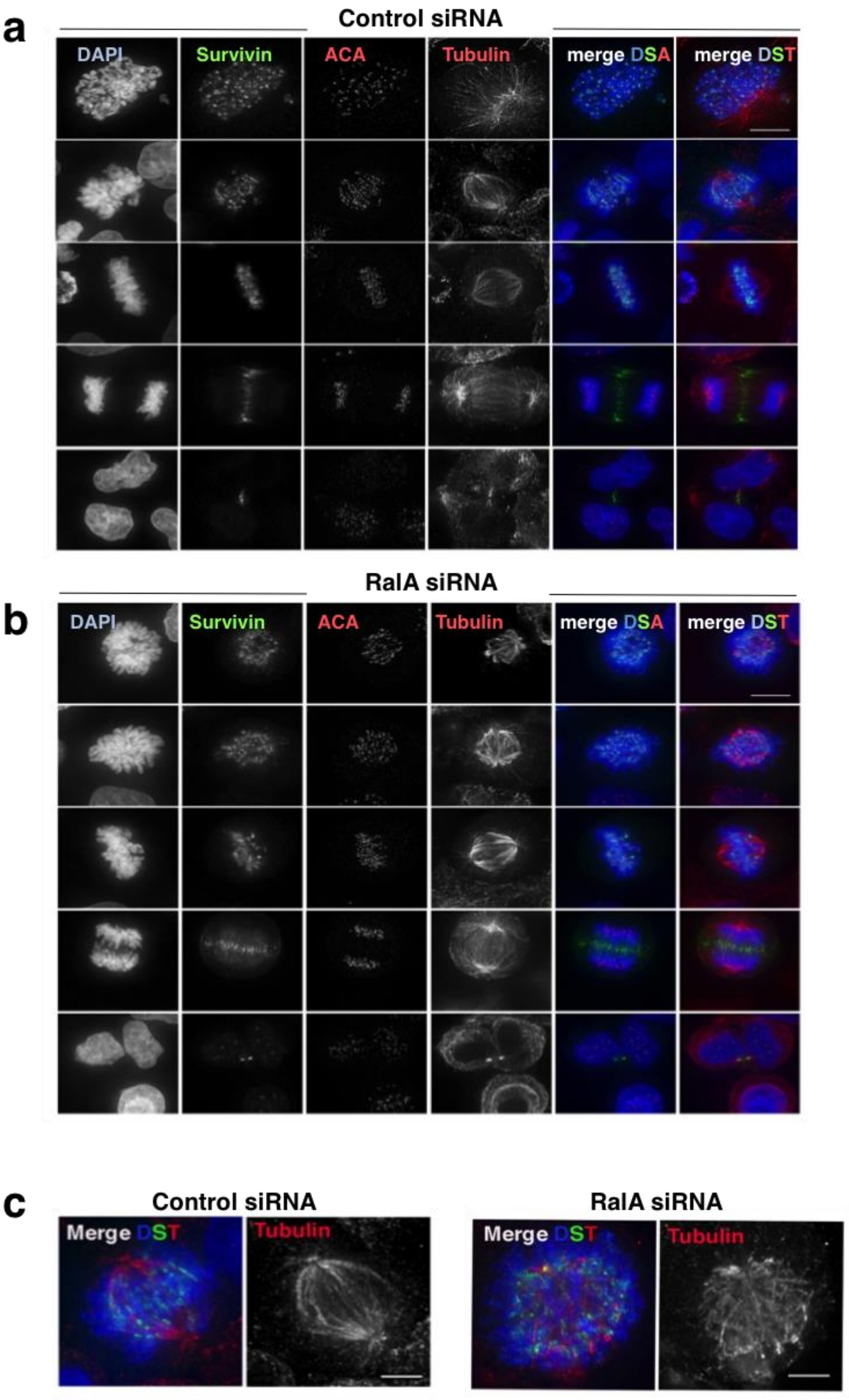

Supplementary Figure 5. Spindle phenotypes following Ral A-depletion.
Knockdown of RalA with siRNA induces an increased frequency of abnormal spindle shapes during prometaphase. As for the TD-60 siRNA, cells seem to subsequently complete mitosis normally. Control siRNA (A) and RalA-depleted cells (B) 72 hours after transfection with siRNA were fixed with 4% PFA, processed for immunofluorescence, and stained with DAPI (blue), α-tubulin (red), ACA (Cy5) and Survivin (green). Bar, 10 μm. **(c)** High magnification of abnormal spindle shapes observed after RalA ablation. Control and RalA-depleted cells fixed with 4% PFA were stained with DAPI (blue), α-tubulin (red) and Survivin (green). Bar, 5 μm.

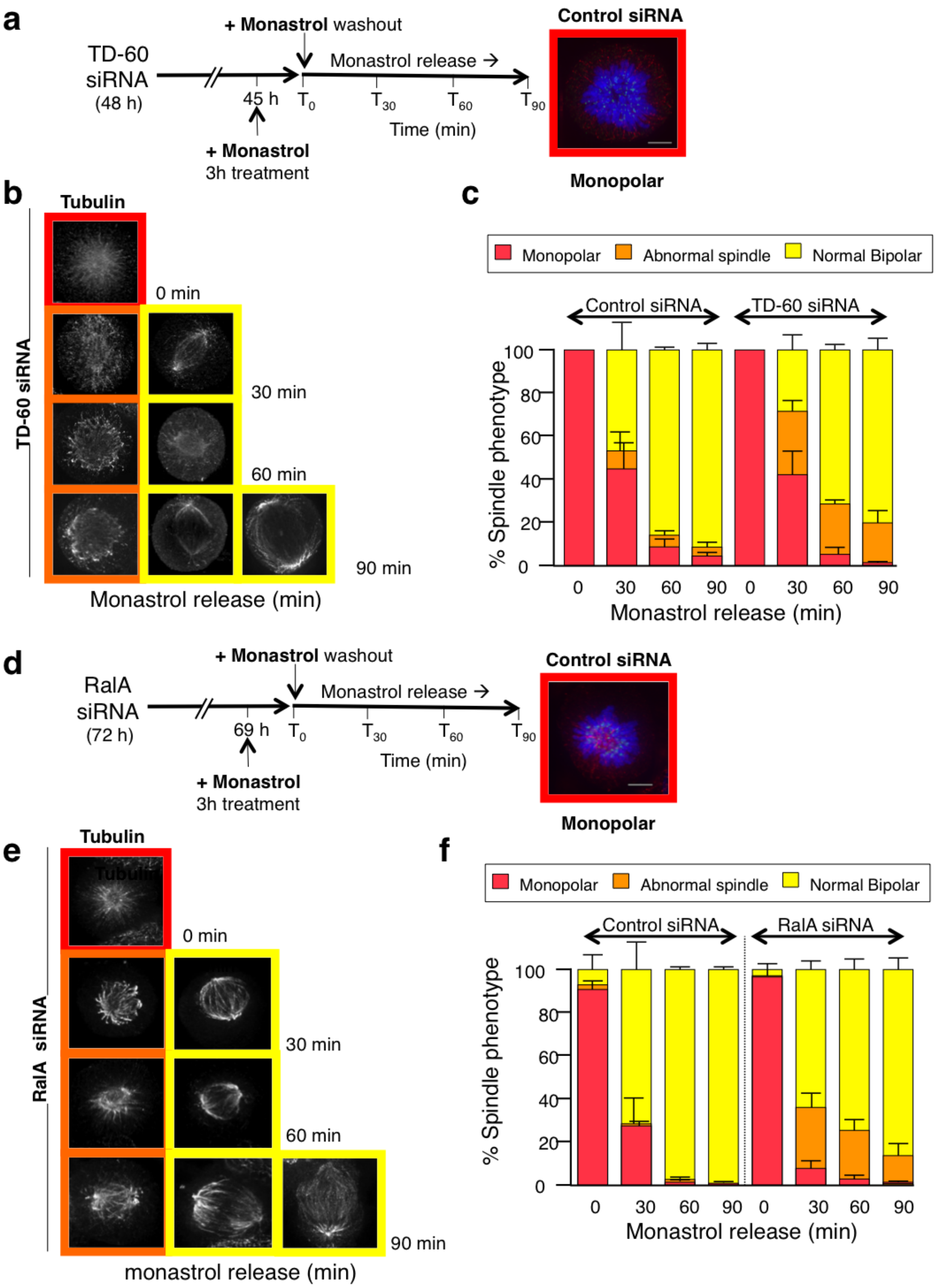

Supplementary Figure 6. TD-60 and RalA depletion both delay spindle recovery from monastrol.
(a) Diagram of the monastrol release experiment for TD-60-depleted cells. **(b)** Representative spindle structures for each time point detected with α-tubulin staining. TD-60 depleted cells were fixed with 4% PFA and stained with DAPI (blue), α-tubulin (red), ACA (green) and Aurora B (Cy5). **(c)** Quantification of mitotic phenotypes for TD-60-depleted cells after monastrol washout at 30, 60 and 90 minutes. Abnormal spindles are similar to those described in the legend of Figure 2d. Graphs report mean ± SEM (3 replicas). **(d)** Diagram of the monastrol release experiment for RalA-depleted cells. **(e)** Representative spindle structures for each time point detected with α-tubulin staining. RalA-depleted cells were fixed with 4% PFA and stained with DAPI (blue), α-tubulin (red), ACA (Cy5) and Aurora B (green). **(f)** Quantification of mitotic phenotypes for RalA-depleted cells after monastrol washout at 30, 60 and 90 minutes. Graphs report mean ± SEM (n=3).

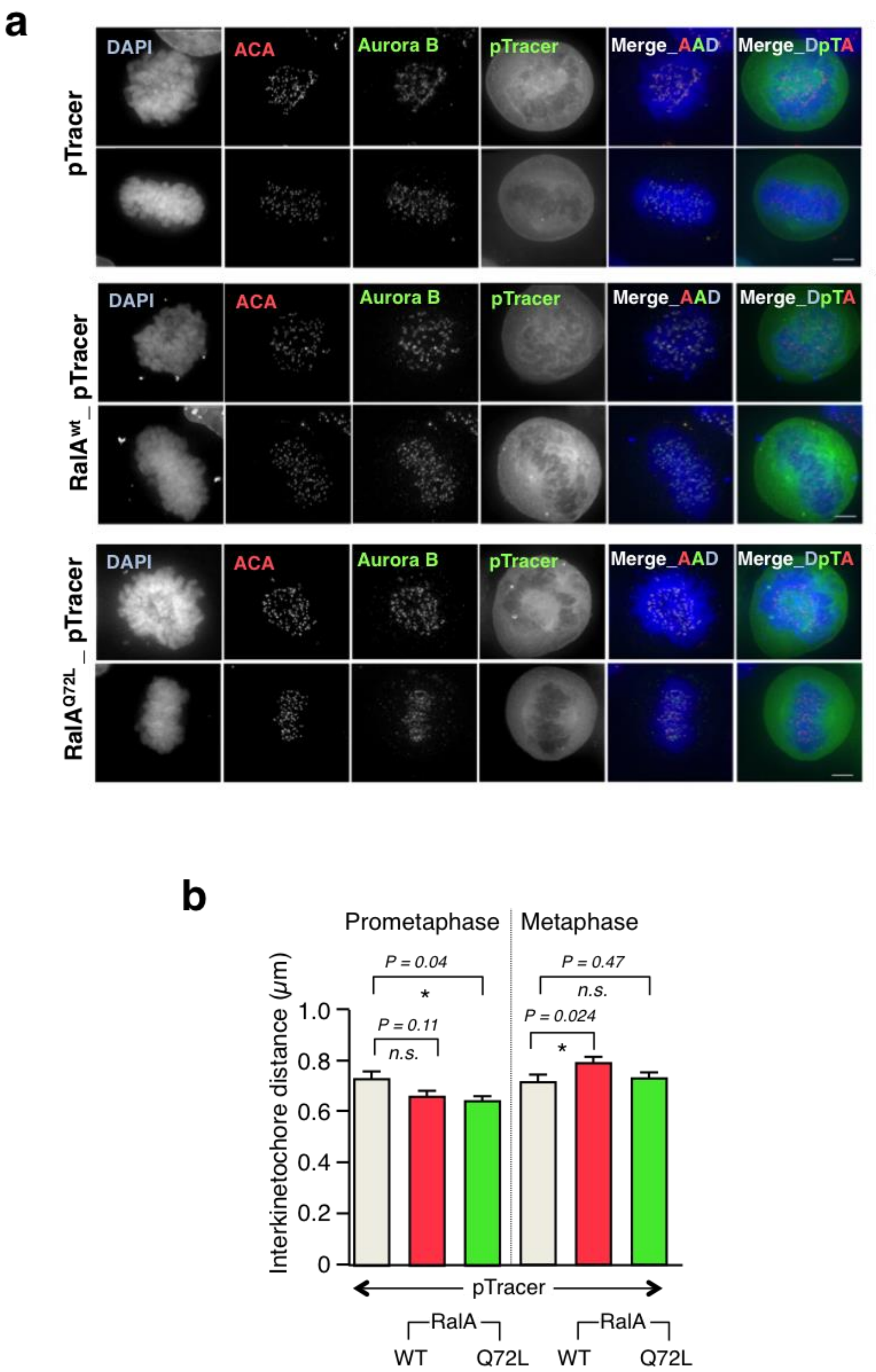

Supplementary Figure 7. Expression of RalA wt and RalAQ72L in HeLa cells in the absence of TD-60 siRNA has only minor effects on kinetochore stretch.

(a) HeLa cells were transfected with Empty pTRACER, RalA wt pTRACER and RalA Q72L pTRACER and stained for DAPI (blue), ACA (red-Cy5) and Aurora B (green). **(b)** Inter-kinetochore distance (µm) was determined using ImageJ.

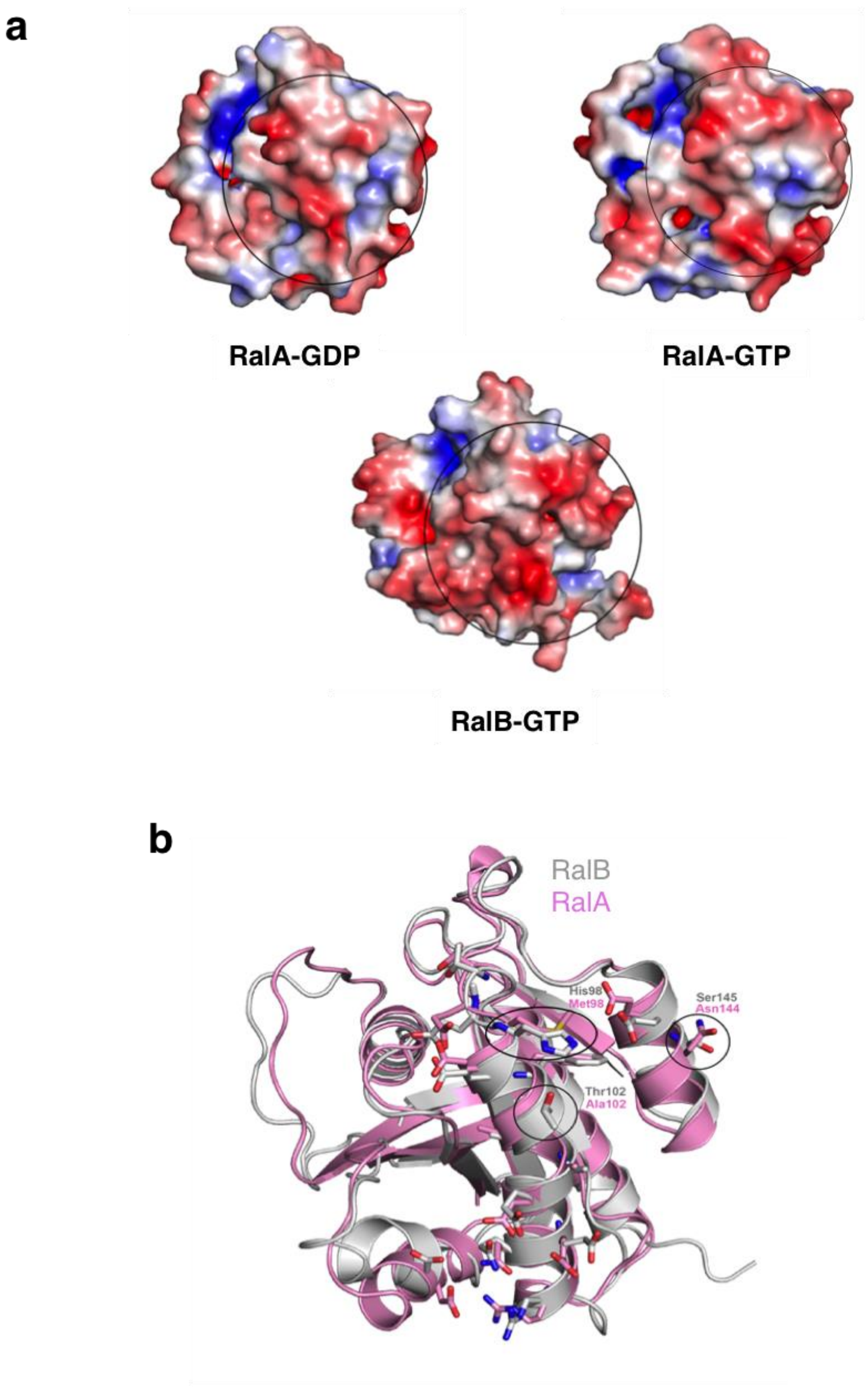

Supplementary Figure 8: RalA and RalB show differences in their structure
(a) Electrostatic surfaces of active RalA ("RalA-GTP"; RalA-GMPPNP from a complex with Exo84; PBD id: 1ZC4), inactive RalA ("RalA-GDP" from a complex with C3bot; PDB id: 2A78) and active RalB ("RalB-GTP"; RalB-GMPPNP from a complex with RLIP76; PDB ID: 2KWI) were calculated using the APBS plug-in in PyMOL (The PyMOL Molecular Graphics System, Version 1.5.0.4 Schrödinger, LLC). **(b)** Superposition of a RalA crystal structure (pink; 1ZC4) using the Phyre 2 server, webserver, http://www.sbg.bio.ic.ac.uk/phyre2[34], onto the NMR structure of RalB (grey; 2KWI) shows that the overall structure of RalA is similar to RalB, but with 3 amino acid residue changes at the predicted interface with TD-60 (highlighted by black circle).

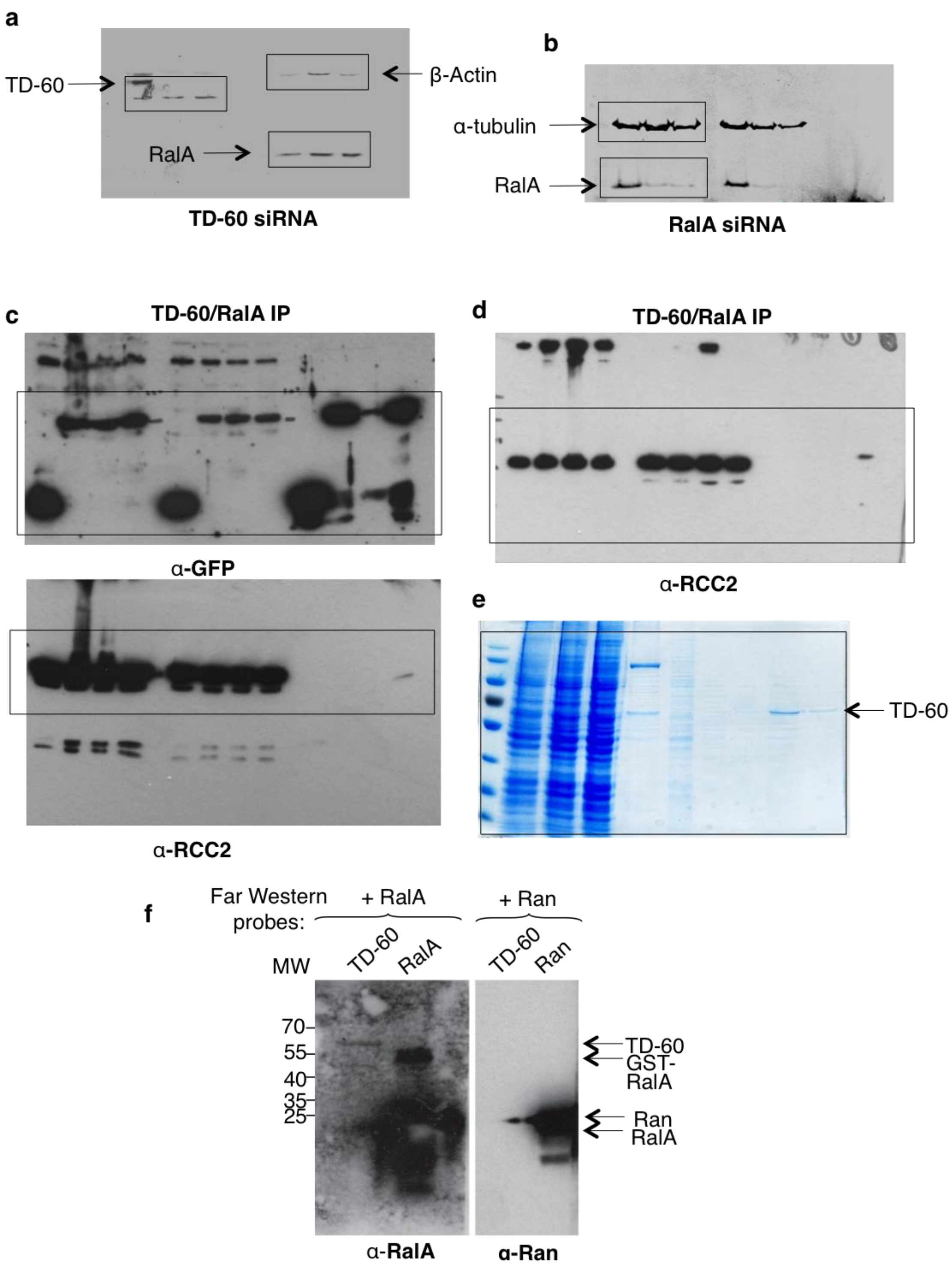

Supplementary Figure 9: Full scans of gels shown in (a) Figure 2a (b) Figure 3a (c) Supplementary Figure 2d (d) Figure 1f (e) Supplementary Figure 1e and (f) Supplementary Figure 2b

Supplementary Table 1: Statistic of TD-60 GDP release of Figure 1b (Unpaired t test P<0.05)

TD-60 GDP release (pmol)

**CTRL GTPase +**	**selected GTPase**	**Significance**	**P value**
RalA/TD-60	Rab5a/Rabex	**n.s.**	0.0578
RalA/TD-60	**Ran/TD-60**	***	**<0.0001**
RalA/TD-60	Rac1/TD-60	**	0.0041
RalA/TD-60	RhoA/TD-60	**	0.0064
RalA/TD-60	Cdc42/TD-60	***	0.0009
RalA/TD-60	Rac2/TD-60	**	0.0011
RalA/TD-60	RhoG/TD-60	*	0.0390
RalA/TD-60	NRas/TD-60	***	0.0009
RalA/TD-60	RalB/TD-60	***	0.0005
RalA/TD-60	KRas2b/TD-60	**	0.0027
RalA/TD-60	Rab4a/TD-60	**	0.0015
RalA/TD-60	Rab11/TD-60	***	0.0006
RalA/TD-60	Rab21/TD-60	**	0.0024
RalA/TD-60	Rab14/TD-60	***	0.0004

Ran family	Ras family	Rho family	Rab family

SUPPLEMENTARY METHODS

TD-60 production

For production of human recombinant SBP-TD-60 protein we used a baculovirus expression system. SBP-TD-60 baculovirus was generated using the "Bac-to-Bac" system (Life Technologies) according to the manufacturer's protocol. SF9 cells were grown in roller bottles on a shaking platform at 100 rpm, at 27°C. 300 ml of culture at 1.0x10^{6} cells/ml were harvested by centrifugation and infected with 20 ml of high MOI TD-60 baculovirus, and incubated for an 1h at 27 °C. Cells were then diluted with 200 ml of SF-900 II medium (Life Technologies) and incubated for 72 h at 27 °C. Cells were pelleted by centrifugation at 1300 x g at 4°C for 5 min and lysed in 50 ml of lysis buffer (50mM Tris pH 8.0, 0.4 M NaCl, 0.5% NP-40, 0.1% deoxycholate (DOC), 200 µM PMSF, 1 mg/ml CLAP, 1 mg/ml Aprotinin). The cell lysate was centrifuged for 15 min at 13,000 x g. The supernatant was collected and added to 3ml of pre-washed and equilibrated (2X in TD-60 lysis buffer) streptavidin agarose beads (Pierce Streptavidin Plus UltraLink Resin) and incubated with the overnight at 4°C on a rotating wheel.

The beads were then collected by centrifugation at 2,500 x g for 5 min and washed three times for 10 - 20 min at 4°C with 15 ml of lysis buffer followed by centrifugation at 2,500 x g for 5 min. Recombinant protein was eluted with two to three washes using 4 ml of elution buffer (50 mM Tris pH 7.4, 250 mM NaCl, 4 mM Biotin, 0.5% NP-40, 0.1% DOC) while incubating for 20 min at 4°C on a rotating wheel. Eluates were dialyzed against elution buffer overnight at 4°C. Alternatively, purified protein was desalted prior to an enzymatic activity assay using a Zeba desalting column (Thermo). Protein purity and quantity was screened by high sensitivity Coomassie Blue staining (Instant Blue – Expedeon).

GTPase production

GTPases proteins for the GEF assay were purified according to purification methods published by the Barr lab in (Yoshimura et al., 2010), (Linford et al., 2012) and (Bastos et al., 2012). A subset of the purified GTPases used is shown in Suppl Figure 1E. Gels of the others are shown in (Linford et al., 2012) and (Bastos et al., 2012). All GTPases migrated as a single band in Coomasie blue-stained SDS-PAGE.

For Far-Western assay, RalA and Ran were cloned into pFAT2 containing a thrombin site between the N-terminal hexa-histidine-GST tag and the multiple cloning site. His-GST fusion proteins were expressed in E. coli BL21 Gold by induction with 0.35mM IPTG overnight at 18°C. Cells were lysed in 20mM Tris, 300mM NaCl, 20mM Imidazole, 2mM β-mercaptoethanol (pH 8.0) containing 1X Complete protease inhibitor cocktail (Roche) and 10μg/ml DNAse I (Sigma) using sonication. Clarified lysates were applied to a Ni-NTA (GE Healthcare) affinity column. The protein-bound columns were washed with high salt buffer (20mM Tris, 1000mM NaCl, 50mM Imidazole, 2mM β-mercaptoethanol, pH8) followed by cleavage buffer (20mM Tris, 300mM NaCl, 50mM Imidazole, 2mM β-mercaptoethanol, pH8) wash, before the proteins were cleaved on the column with thrombin protease (GE Healthcare, 10units/mg of protein) overnight at 4°C. Thrombin cleavable proteins were eluted with cleavage buffer. Subsequently,

proteins were further purified by size exclusion chromatography in 20mM Tris, 100mM NaCl, 2mM DTT, pH8.0 (Superdex 75 10/300 GL, GE Healthcare).

Far Western analysis

10 µl of purified SBP-TD-60 were loaded in triplicate on SDS-PAGE and transferred on a PVDF membrane, which was blocked with 5% Milk-TBS + 0.05% Tween. Individual PVDF strips with SBP-TD-60 was probed with 6µg/µl of purified cleaved-RalA or Ran in 5% Milk-TBS-T overnight at 4°C. As negative control, an extra PVDF strip with SBP-TD-60 was probed with only blocking solution overnight at 4°C. Following 3 washes with 5% Milk-TBS + 0.05% Tween, the membranes were immunoblotted with either anti-RalA and anti-Ran mouse antibody (BD) for 2 hours at RT, and HRP-conjugated anti-mouse secondary antibody (GE Amersham) for 1hour at RT. Secondary antibodies were detected by ECL.

Monastrol recovery assay

HeLa cells were transfected with Control and TD-60 or RalA siRNA double–stranded oligos and processed for a monastrol release experiment. 100 nM monastrol was added at 45 hours after siRNA transfection for TD-60 RNAi and at 69 hours for RalA RNAi. Monastrol was washed out at time 0. Cells were analyzed at the following time points: 30, 60 and 90 minutes.
